# Using a targeted metabolomics approach to explore differences in ARDS associated with COVID-19 compared to ARDS caused by H1N1 influenza and bacterial pneumonia

**DOI:** 10.1186/s13054-024-04843-0

**Published:** 2024-02-27

**Authors:** Chel Hee Lee, Mohammad M. Banoei, Mariam Ansari, Matthew P. Cheng, Francois Lamontagne, Donald Griesdale, David E. Lasry, Koray Demir, Vinay Dhingra, Karen C. Tran, Terry Lee, Kevin Burns, David Sweet, John Marshall, Arthur Slutsky, Srinivas Murthy, Joel Singer, David M. Patrick, Todd C. Lee, John H. Boyd, Keith R. Walley, Robert Fowler, Greg Haljan, Donald C. Vinh, Alison Mcgeer, David Maslove, Puneet Mann, Kathryn Donohoe, Geraldine Hernandez, Genevieve Rocheleau, Uriel Trahtemberg, Anand Kumar, Ma Lou, Claudia dos Santos, Andrew Baker, James A. Russell, Brent W. Winston, for the *ARBs CORONA I. Investigators, J. A. Russell, K. R. Walley, J. Boyd, T. Lee, J. Singer, D. Sweet, K. Tran, S. Reynolds, G. Haljan, M. Cheng, D. Vinh, T. Lee, F. Lamontagne, B. Winston, O. Rewa, J. Marshall, A. Slutsky, A. McGeer, V. Sivanantham, R. Fowler, D. Maslove, S. Perez Patrigeon, K. D. Burns

**Affiliations:** 1 Department of Critical Care Medicine, University of Calgary, Alberta, Canada; 2 Divisions of Infectious Diseases & Medical Microbiology, McGill University Health Center, McGill’s Interdisciplinary Initiative in Infection and Immunity, Montreal, PQ Canada; 3 University of Sherbrooke, Sherbrooke, QC Canada; 4 Critical Care Medicine, Vancouver General Hospital and University of British Columbia, 2775 Laurel St, Vancouver, BC V5Z 1M9 Canada; 5 Division of General Internal Medicine, Vancouver General Hospital and University of British Columbia, 2775 Laurel St, Vancouver, BC V5Z 1M9 Canada; 6 Centre for Health Evaluation and Outcome Science (CHEOS), St. Paul’s Hospital and University of British Columbia, 1081 Burrard Street, Vancouver, BC V6Z 1Y6 Canada; 7 Department of Medicine, Division of Nephrology, Ottawa Hospital Research Institute, and University of Ottawa, 1967 Riverside Dr., Rm. 535, Ottawa, ON K1H 7W9 Canada; 8 Critical Care Medicine and Emergency Medicine, Vancouver General Hospital and University of British Columbia, 2775 Laurel St, Vancouver, BC V5Z 1M9 Canada; 9 Department of Surgery, St. Michael’s Hospital and University of Toronto, 30 Bond Street, Toronto, ON M5B 1W8 Canada; 10 Keenan Research Centre for Biomedical Science, Li Ka Shing Knowledge Institute, St. Michael’s Hospital; Interdepartmental Division of Critical Care Medicine, University of Toronto, Toronto, Canada; 11 British Columbia Children’s Hospital, University of British Columbia, 4500 Oak Street, Vancouver, BC V6H 3N1 Canada; 12 British Columbia Centre for Disease Control (BCCDC) and School of Population and Public Health, University of British Columbia, 655 West 12th Avenue, Vancouver, BC V5Z 4R4 Canada; 13 Centre for Heart Lung Innovation, St. Paul’s Hospital, University of British Columbia, Vancouver, BC Canada; 14 Division of Critical Care Medicine, St. Paul’s Hospital, University of British Columbia, Vancouver, BC Canada; 15 Sunnybrook Health Sciences Centre, 2075 Bayview Avenue, Toronto, ON M4N 3M5 Canada; 16 Departments of Critical Care Medicine, Medicine and Biochemistry and Molecular Biology, University of Calgary, Health Research Innovation Center (HRIC), Room 4C64, 3280 Hospital Drive NW, Calgary, AB T2N 4Z6 Canada; 17 Department of Medicine and Critical Care Medicine, Surrey Memorial Hospital, 13750 96th Avenue, Surrey, BC V3V 1Z2 Canada; 18 Mt. Sinai Hospital and University of Toronto, 600 University Avenue, Toronto, ON M5G 1X5 Canada; 19 Department of Critical Care, Kingston General Hospital and Queen’s University, 76 Stuart Street, Kingston, ON K7L 2V7 Canada; 20 Black Tusk, Vancouver, BC Canada; 21 Department of Critical Care, Galilee Medical Center, Nahariya, Israel; 22 Bar Ilan University, Ramat Gan, Israel; 23 Keenan Research Centre for Biomedical Science, Li Ka Shing Knowledge Institute, St. Michael’s Hospital, Toronto, Canada; 24 Departments of Medicine and Medical Microbiology, University of Manitoba, Winnipeg, Canada; 25 Department of Medicine and Interdepartmental Division of Critical Care, University of Toronto, Toronto, Canada; 26 Departments of Critical Care and Anesthesia, St. Michael’s Hospital, University of Toronto, Toronto, ON Canada; 27 St. Paul’s Hospital [Coordinating Centre], Vancouver, BC Canada; 28 Vancouver General Hospital, Vancouver, BC Canada; 29 Royal Columbian Hospital, Vancouver, BC Canada; 30 Surrey Memorial Hospital, Surrey, BC Canada; 31 McGill University Centre Hospital, Montreal, QC Canada; 32 Jewish General Hospital, Montreal, QC Canada; 33 University de Sherbrooke, Sherbrooke, QC Canada; 34 Foothills Medical Centre, Calgary, AB Canada; 35 University of Alberta, Edmonton, AB Canada; 36 St. Michael’s Hospital, Toronto, ON Canada; 37 Mount Sinai Hospital, Toronto, ON Canada; 38 Sunnybrook and Women’s College Health Science Centre, Toronto, ON Canada; 39 Kingston General Hospital, Kingston, ON Canada; 40 Ottawa Hospital Research Institute, University of Ottawa, Ottawa, ON Canada

**Keywords:** Acute respiratory distress syndrome, SARS-CoV-2, H1N1, Pneumonia, Metabolomics

## Abstract

**Rationale:**

Acute respiratory distress syndrome (ARDS) is a life-threatening critical care syndrome commonly associated with infections such as COVID-19, influenza, and bacterial pneumonia. Ongoing research aims to improve our understanding of ARDS, including its molecular mechanisms, individualized treatment options, and potential interventions to reduce inflammation and promote lung repair.

**Objective:**

To map and compare metabolic phenotypes of different infectious causes of ARDS to better understand the metabolic pathways involved in the underlying pathogenesis.

**Methods:**

We analyzed metabolic phenotypes of 3 ARDS cohorts caused by COVID-19, H1N1 influenza, and bacterial pneumonia compared to non-ARDS COVID-19-infected patients and ICU-ventilated controls. Targeted metabolomics was performed on plasma samples from a total of 150 patients using quantitative LC–MS/MS and DI-MS/MS analytical platforms.

**Results:**

Distinct metabolic phenotypes were detected between different infectious causes of ARDS. There were metabolomics differences between ARDSs associated with COVID-19 and H1N1, which include metabolic pathways involving taurine and hypotaurine, pyruvate, TCA cycle metabolites, lysine, and glycerophospholipids. ARDSs associated with bacterial pneumonia and COVID-19 differed in the metabolism of D-glutamine and D-glutamate, arginine, proline, histidine, and pyruvate. The metabolic profile of COVID-19 ARDS (C19/A) patients admitted to the ICU differed from COVID-19 pneumonia (C19/P) patients who were not admitted to the ICU in metabolisms of phenylalanine, tryptophan, lysine, and tyrosine. Metabolomics analysis revealed significant differences between C19/A, H1N1/A, and PNA/A vs ICU-ventilated controls, reflecting potentially different disease mechanisms.

**Conclusion:**

Different metabolic phenotypes characterize ARDS associated with different viral and bacterial infections.

**Supplementary Information:**

The online version contains supplementary material available at 10.1186/s13054-024-04843-0.

## Introduction

COVID-19 has had an enormous global impact, affecting millions of people, causing many deaths, and still requiring a great effort to understand the mechanism of COVID-19 disease better. SARS-CoV-2 acts similarly to H1N1, the disease caused by Influenza A type virus [1]. Although they utilize different receptors for viral entry, SARS-CoV-2 using the angiotensin-converting enzyme type 2 (ACE2) receptor and H1N1 using sialic acid receptors, they have both been implicated in affecting the renin–angiotensin–aldosterone system (RAAS) pathway [2–4] and have been shown to cause ARDS [4–7].

In severe forms, COVID-19 and H1N1 can result in acute respiratory distress syndrome (ARDS), leading to the development of multiorgan damage [4–7]. Mortality rates for patients with ARDS are as high as 38%, with no specific ARDS pharmacologic therapy proven to date [8]. Despite this, early non-specific therapy has improved outcomes, illustrating the importance of timely diagnosis [9]. Few diagnostic biomarkers have been proposed, found, or validated for ARDS.

Metabolomics studies can help reveal altered metabolic pathways during COVID-19 infection (as well as other viral and bacterial infections) and the development of ARDS, providing insight into the disease processes. In addition, it provides an opportunity to investigate how SARS-CoV-2 affects the host's metabolism and immune response. Most metabolomics studies involving COVID-19 compare SARS-CoV-2-infected patients with normal controls and focus on differentiating severity. However, few studies have been done comparing ARDS caused by different etiologies [10–12]. Overall, perturbed pathways currently observed in COVID-19 include pyruvate metabolism, kynurenine pathways, and amino acid metabolism—specifically tryptophan metabolism [13–19].

We aimed first to compare the metabolomic profiles between different infectious etiologies of ARDS: COVID-19-associated ARDS (C19/A), bacterial pneumonia-associated ARDS (PNA/A), and H1N1-associated ARDS (H1N1/A). We additionally sought to compare the metabolomic profiles of patients with COVID-19 ARDS admitted to the ICU to those admitted to the hospital but not requiring ICU admission (i.e., those with COVID-19 pneumonia but not severe enough to require ICU admission). Subsequently, we sought to propose a bedside formula by identifying the minimal metabolites associated with the mechanisms differentiating these groups for early diagnosis of COVID-19 ARDS vs other infectious causes of ARDS and for COVID-19 severity assessment of patients.

## Materials and methods

### Data sources and measurements

We collected plasma samples from four different tissue banks in Canada. All samples were plasma collected, isolated, and managed in a similar fashion. Each study group consisted of 25 patients with plasma samples drawn within 24 h of ICU admission for ARDS patients and within 24 h of hospital admission for COVID-19 pneumonia (C19/P) patients not sick enough to be admitted to the ICU. C19/A is a group of COVID-19-infected (PCR-positive) ICU patients with ARDS who were ventilated on the first day of ICU admission. C19/P is a group of COVID-19-infected (PCR positive) non-ICU hospitalized pneumonia patients on the first day of admission to the hospital. PNA/A is a group of non-COVID-19, bacterial pneumonia-associated (culture-positive) ARDS patients ventilated on the first day of ICU admission. H1N1/A is a group of non-COVID-19, H1N1-associated (PCR positive) ARDS patients ventilated on the first day of ICU admission. Finally, the CTL group consisted of a group of patients not suspected of having pneumonia (viral or bacterial) mechanically ventilated ICU controls who were either postoperative patients included in the study with samples taken while ventilated in the ICU 6–24 h following major cardiovascular surgery, such as coronary artery bypass graft (CABG), or patients with severe neurological diseases, such as stroke, subarachnoid hemorrhage, or meningitis without pneumonia, with samples taken within 24 h of ICU admission while intubated and ventilated.

C19/A and C19/P samples were collected as part of the ARBs CORONA I multicenter study [20, 21]. CTL and PNA/A samples were collected at Foothills Medical Center and Peter Lougheed Center (Calgary, AB, Canada) during the period 2009–2014 and processed similarly (as published in the Canadian Critical Care Translational Biology Group website protocols) and stored at −80 °C at the University of Calgary as part of the CCEPTR ICU tissue bank. H1N1/A samples were collected during the H1N1 pandemic in 2009 and processed identically to the CCCTBG website protocol. They were stored at −80 °C and made available from Winnipeg, Manitoba. In addition, C19/AV, a validation group of COVID-19-associated ARDS, consisted of 25 patients with plasma samples collected under identical conditions as the C19/A samples from the ARBs Corona I study (i.e., patients with COVID-19-associated ARDS (PCR positive) admitted to the ICU with samples taken within 24 h of ICU admission) but are from the University of Toronto and the University of Calgary. These samples were processed identically to the CCCTBG and CCEPTR protocols and stored in aliquots at −80 °C.

The Berlin definition was used for ARDS diagnosis. Two investigators verified the diagnosis for the bacterial pneumonia-associated ARDS in particular—Dr. Brent Winsrton and an MD, Ph.D. student, Dr. Sayed Metwaly, from a previously published study (Metwaly, S. et al. "ARDS Metabolic Fingerprints: Characterization, Benchmarking, and Potential Mechanistic Interpretation." Am J Physiol Lung Cell Mol Physiol. 2021 May 5, 321: L79–L90. doi, 10.1152/ajplung.00077.2021.) The diagnosis of ARDS in the H1N1 cohort was made by Dr. Anand Kumar and Dr. Brent Winston, and Dr. Brent Winston verified the ARDS diagnosis in the COVID-19 cohort. We collected clinical information such as age, sex, PaO_2_/FiO_2_, type of COVID-19 medication used (e.g., steroids or Remdesivir), ventilation support, COVID-19 test result, H1N1 test result, bacterial culture result, survival status at 28 days from hospital admission and ICU and hospital discharge.

### Study design

All study groups are matched by age and sex. Patients were chosen randomly from each cohort if they matched age and sex, and plasma samples were available. Age matching was done ± 5 years. Four groups (C19/A, PNA/A, H1N1/A, and CTL) underwent quantitative metabolomics analysis (as described above) followed by multiple and pairwise comparisons of the metabolite findings. We started with characterizing the metabolomic profile of each group using all metabolites included in the study and ran simultaneous comparisons of their profiles. We then ran six pairwise comparisons as follows. The three pairwise comparisons, each with CTL as a reference group and the other ARDS groups for direct comparison, allowed us to see how the specific ARDS subgroups deviated from the ICU-ventilated control group (CTL) regarding metabolomic profile. In addition, the other three pairwise comparisons informed us of how different the infectious-mediated ARDS groups are. To assess the severity of COVID-19 patients, we compare C19/A to C19/P (the C19/P patients had COVID-19 pneumonia but not ARDS and were not severe enough to be admitted to the ICU) with plasma samples taken on day one after hospital admission for C19/P or day one after ICU admission for C19/A. Finally, plasma metabolomics of a validation cohort for COVID-19 ARDS (C19/AV) patient samples was compared to C19/A patient samples to validate our COVID-19 ARDS findings. The Conjoint Health Research Ethics Board, University of Calgary, has reviewed and approved this study (Ethics ID: REB20-0654). We used 25 patients per cohort based on a previous study [22].

### Sample preparation

For organic acid quantification, 50 µl plasma samples were thawed on ice, followed by adding 150 µl of ice-cold methanol and 10 µl of isotope-labeled standards. The mixtures were kept overnight at −20 °C to precipitate proteins, followed by centrifugation at 13,000 × g for 20 min. A total of 50 µl of supernatant of the extracts were added to the center of a 96-well plate, followed by adding a 3-nitrophenylhydrazine reagent to the extract and incubated for two hours. Butylated hydroxytoluene (2 mg/ml) stabilizer and water were added to the extract before LC–MS/MS injection.

For amino acid and lipid quantifications, samples were vortexed and centrifuged, adding 10 µl of samples to a 96-well plate and a stream of nitrogen-dried samples. Phenyl-isothiocyanate reagent was added to the samples in the plate. Samples were incubated and then dried using an evaporator. Three hundred microliters of extraction solvent was added to the analytes. Extracts were centrifuged to the lower part of the 96-well plate; a dilution step was performed using 0.2% formic acid in the water and 0.2% formic acid in acetonitrile.

### Metabolomics profiles

Plasma-based targeted metabolomics was performed to quantify the concentration of 143 metabolites developed by The Metabolomics Innovation Center (TMIC) at the University of Alberta, Edmonton (see list of metabolites in the supplement) [16, 23] and as we have previously done [24]. Reverse-phase liquid chromatography-tandem mass spectrometry (LC–MS/MS) was applied to analyze amino acids, biogenic amines, and organic acids. Direct infusion tandem mass spectrometry (DI-MS/MS) was applied to quantify glycerophospholipids, lysophosphatidylcholines (lysoPCs), and phosphatidylcholines (PCs), acylcarnitines (Cs), and sphingomyelins (SMs). Mass spectrometry was analyzed using an ABSciex 4000 Qtrap tandem MS instrument (Applied Biosystems/MDS Analytical Technologies, Foster City, CA, USA). An Agilent 1260 series UHPLC system (Agilent Technologies, Palo Alto, CA) was combined with MS for LC–MS/MS [16, 23].

### LC–MS/MS analyses

For chromatography, an Agilent reversed-phase Zorbax Eclipse XDB C18 column (3.0 mm × 100 mm, 3.5 μm particle size, 80 A pore size) with a Phenomenex (Torrance, CA, USA) Security Guard C18 pre-column (4.0 mm × 3.0 mm) was used for analyzing amino acids and biogenic amines. The parameters for LC–MS/MS analysis were as follows: Mobile phase A was 0.2% (v/v) formic acid in the water, and mobile phase B was 0.2% (v/v) formic acid in acetonitrile. The gradient parameters were *t* = 0 min, 0% B; *t* = 0.5 min, 0% B; *t* = 5.5 min, 95% B; *t* = 6.5 min, 95% B; *t* = 7.0 min, 0% B; and *t* = 9.5 min, 0% B. The chromatography column was set as 50 ºC. Ten microliters of samples was injected into the column with a flow rate of 300 µl/min.

For chromatography of organic acids, mobile phase A was 0.01% (v/v) formic acid in the water, and mobile phase B was 0.01% (v/v) formic acid in methanol. The gradient parameters were *t* = 0 min, 30% B; *t* = 2.0 min, 50% B; *t* = 12.5 min, 95% B; *t* = 12.5 min, 100% B; *t* = 13.5 min, 100% B; and *t* = 13.6 min, and finally 30% B for 4.4 min. The chromatography column was set as 40 ºC. Ten microliters of samples was injected into the column with a flow rate of 300 µl/min.

### DI-MS/MS analysis

The direct infusion was performed using the connection of the LC autosampler to the MS ion source using red PEEK tubing. The mobile phase was set by mixing 60 µl of formic acid, 10 ml of water, and 290 ml of methanol. The flow rate was *t* = 0 min, 30 µl/min; *t* = 1.6 min, 30 µl/min; *t* = 2.4 min, 200 µl/min; *t* = 2.8 min, 200 ul/min' and *t* = 3.0 min, 30 µl/min. Twenty microliters of samples was injected into the MS.

### Quantification of metabolites

A seven-point standard calibration curve was obtained for each metabolite to quantify organic acids, amino acids, and biogenic amines. The signal intensity of each metabolite was corrected to the corresponding isotope-labeled internal standard, and the known concentrations were calculated based on the quadric regression with a 1/ × 2 weighting. The concentrations of lipids and glucose were calculated semi-quantitatively using a single-point calibration of representative metabolites built based on the same class of compound with the same core structure, assuming a linear regression through zero. Analyst 1.6.2 and MultiQuant 3.0.3 were used to analyze all metabolites in the assay.

### Metabolic phenotyping

Both LC–MS/MS and DI-MS/MS analytical platforms were applied in a targeted approach to quantify 143 metabolites, including different metabolite classes. Mass spectrometry analysis was performed using an ABSciex 4000 Qtrap tandem MS instrument (Applied Biosystems/MDS Analytical Technologies, Foster City, CA, USA). An Agilent 1260 series UHPLC system (Agilent Technologies, Palo Alto, CA) was combined with MS for LC–MS/MS.[16, 23].

### Statistical analysis and validation

As previously done [24], we processed the raw metabolite concentration data with median-fold normalization, logarithm transformation, and z-score standardization to identify outliers, stabilize variability, and give metabolites an equal contribution weight for model determination (i.e., we normalized the raw data as a standard processing procedure for metabolomics data). Partial least-squares discriminant analysis (PLS-DA) was used as a major analytical model because of the multicollinearity in high-dimensional metabolomic data. A model fitted to the data was assessed by three metrics—R^2^Y (the amount of variance explained by a model of fit), Q^2^Y (cross-validated R^2^, a measure of goodness of prediction of the model), and response permutation test (for validity and to prevent overfitting). The performance of a model was discussed using sensitivity (Se), specificity (Sp), and area under the receiver operator characteristics curve (AUROC). We defined a metabolite selection rule by considering (1) the variable importance of a projection (VIP) score > 1.0 from PLS-DA, (2) the absolute value of logarithm with base 2 of fold change > 1, (3) nonzero coefficients from a penalized logistic regression. The number of latent variables was identified by threefold cross-validation when the PLS-DA model was run. This selection was based on 1000 resamples. All analyses were carried out using a standard statistical computing language and environment, R-4.0.0. The Kyoto Encyclopedia of Genes and Genomes (KEGG) was employed to understand chemical classes and biological pathways. The metabolites with VIP > 1 were projected onto their corresponding KEGG pathways using MetaboAnalyst.

## Results

### Study groups

A total of 150 patients from six groups were enrolled in this study. As shown in Table 1, the mean age is 63 y with a standard deviation of 13, and 42% of patients were female across the groups. In addition, the median length of ICU stay for the ICU groups was 10.0 days with IQR [6, 17] days. The COVID-19 ARDS patient group (C19/A) shows more use of vasopressors (100%), more frequent septic shock (44%), and higher mortality (44%) compared to other study groups (H1N1/A, PNA/A, and CTL in particular).

**Table 1:** Comparison of the study groups' demographic, biochemical, and clinical characteristics. Standard deviations and proportions are given in parentheses for continuous and discrete measures

		C19/A *N* = 25	C19/P *N* = 25	H1N1/A *N* = 25		CTL *N* = 25		PNA/A *N* = 25		C19/AV *N* = 25		Overall *N* = 150
Age, mean (SD)		63 (13)	67 (18)	59.0 (13)		63 (13)		63 (13)		63 (13)		63 (14)
Male sex, n(%)		16 (64%)	10 (40%)	16 (64%)		16 (64%)		16 (64%)		13 (52%)		87 (58%)
BMI, mean (SD)		–	–	34 (11)		28 (5)		33 (9)		30 (9)		32 (9)
Use of vasopressors, n(%)_		25 (100%)	0 (0%)	10 (40%)		14 (56%)		18 (72%)		22 (88%)		89 (60%)
Shock, n(%)		11 (44%)	0 (0%)	5 (20%)		0 (0%)		3 (12%)		3 (12%)		22 (15%)
Comorbidity conditions												
1		7 (28%)	3 (12%)	0 (0%)		4 (16%)		9 (36%)		4(16%)		27 (18%)
2+		18 (72%)	15 (60%)	22(88%)		0 (0%)		11(44%)		7(28%)		73 (49%)
0		0 (0%)	7 (28.0%)	3 (12.0%)		21(84.0%)		5 (20.0%)		14 (56.0%)		50 (34%)
WBC		9 (50)	10 (6)	16 (13)		13 (6)		15 (12)		13 (8)		12.3 (9)
Platelet		242 (80)	240 (111)	218 (166)		181 (52)		221 (151)		248 (94)		225 (116)
Bilirubin		7 (1)	19 (27)	15 (15)		8 (5)		23 (33)		12 (5)		15.7 (22)
Creatine		139 (150)	125 (123)	105 (71)		89 (90)		137 (103)		139 (225)		122 (136)
PF ratio		128 (49)	-	164 (82)		253 (83)		166 (91)		108 (45)		173 (92)
SaO2		85 (14)	94 (4)	96 (3)		96 (2)		91(9)		93 (4)		93 (8)
Medication												
0		0 (0%)	0 (0%)	0 (0%)		25 (100%)		25 (100%)		4 (16%)		54 (36%)
1		1 (4%)	1 (4%)	0 (0%)		0 (0%)		0 (0%)		1 (4%)		3 (2%)
2		1 (4%)	6 (24%)	0 (0%)		0 (0%)		0 (0%)		0 (0%)		7 (5%)
3		23 (92%)	18 (72%)	25 (100%)		0 (0%)		0 (0%)		20 (80%)		86 (57%)
ICU length of stay, mean/median [IQR]		14 [10, 22]	–	10 [7, 14]		3 [2, 4]		11 [7, 16]		17 [10, 46]		10 [6, 17]
Hospital Length of Stay		18 [12, 24]	8 [5, 11]	15 [11, 27]		14 [9, 25]		22 [12, 47]		27 [16, 67]		15 [10, 29]
Death at 28 days		11 (44%)	4 (16%)	8(32%)		0 (0%)		6 (24%)		5 (20%)		34 (23%)

C19/A = SARS-CoV-2-induced ARDS patients admitted to ICU, C19/P = SARS-CoV-2 ARDS patients admitted to hospital not admitted to ICU, H1N1/A = H1N1-induced ARDS patients admitted to ICU, CTL = non-ARDS ventilated control patients admitted to ICU, PNA/A = bacterial pneumonia-induced ARDS patients admitted to ICU, C19/AV = validation group Covid-19-positive patients, admitted to the ICU, equivalent to the C19/A cohort. In medication, 0 = Not received, 1 = Candesartan, 2 = Irbesartan, 3 = Other. Data are from day one of study entry (day one of hospitalization for non-ICU patients or day one of ICU admission)

### Overall difference of metabolomic profiles between groups

**Fig. 1:**
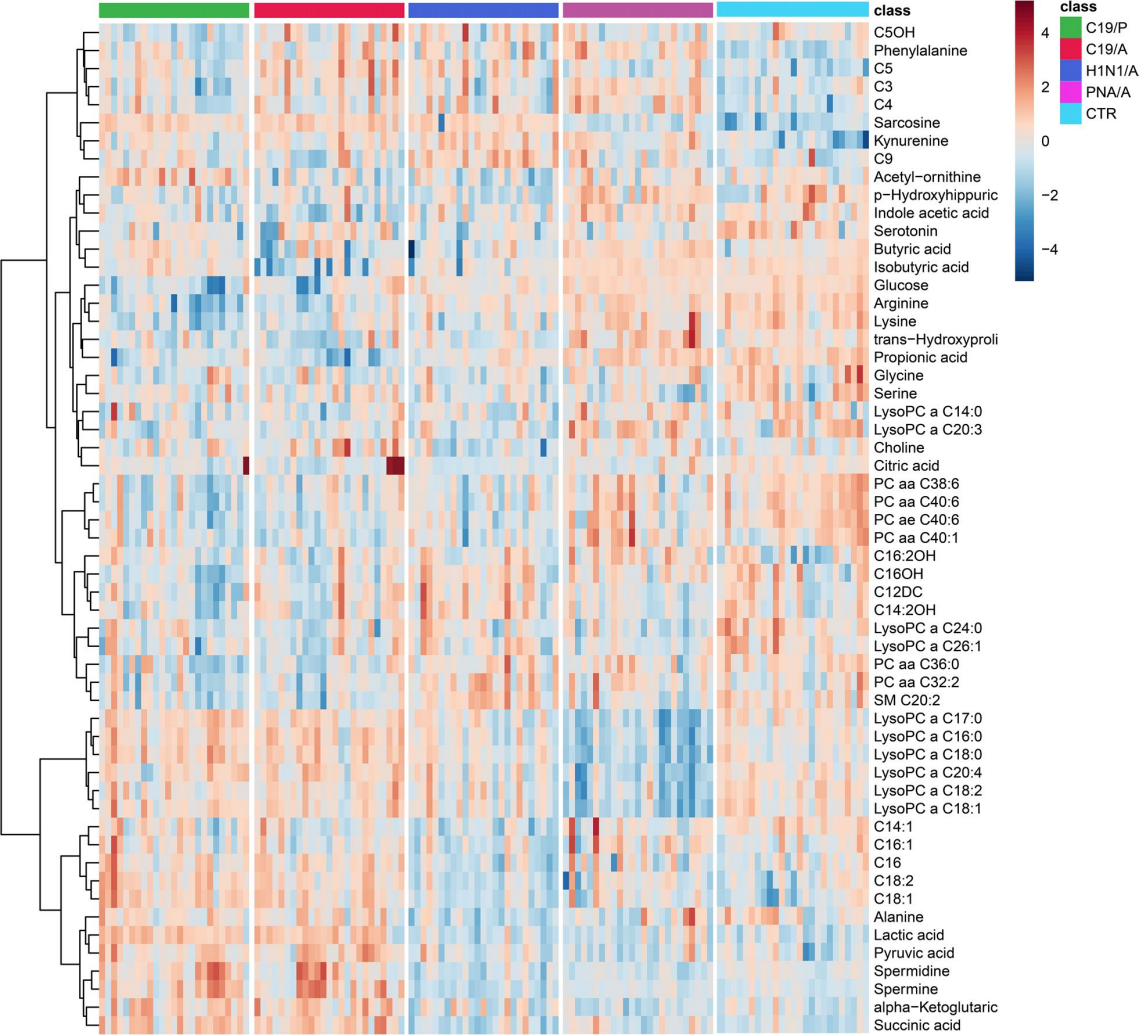
The heatmap shows the difference in metabolomic profile using the most differentiating metabolites (VIP > 1.0) over five study groups. C19/A = SARS-CoV-2-induced ARDS patients admitted to ICU, C19/P = SARS-CoV-2-infected patients not sick enough to be admitted to ICU, H1N1/A = H1N1-induced ARDS patients admitted to ICU, PNA/A = bacterial pneumonia-induced ARDS patients admitted to ICU, CTL = non-ARDS ventilated control patients admitted to ICU. Relative metabolite concentrations are shown as indicated by a scale between −4 and + 4

**Fig. 2:**
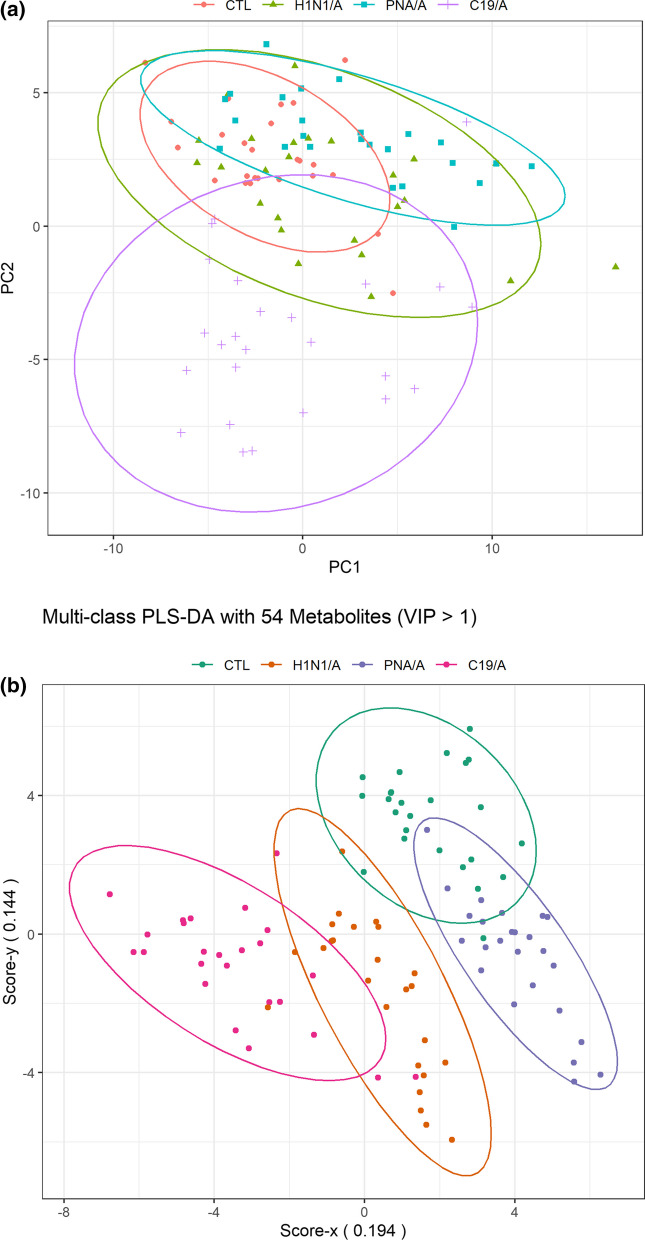
**A** A PCA (principal component analysis) score plot of the first two principal components (PC1 and PC2). Patients are grouped by causes of ARDS indicated by the labels (and color). **B** The score plot from a multi-class PLS-DA model. Patients are grouped by causes of ARDS (indicated by label and color) using the metabolomic signature. The signature consists of 54 metabolites whose variable importance score on projection is greater than 1. The metabolites are listed in Table 2. A model is run with 25 patients in each group. C19/A = COVID-19-induced ARDS patients admitted to ICU, H1N1/A = H1N1-induced ARDS patients admitted to ICU, CTL = non-ARDS ventilated control patients admitted to ICU, PNA/A = bacterial pneumonia-induced ARDS patients admitted to ICU. VIP = variable importance on projection

We did an exploratory data analysis using a heatmap and observed distinct metabolomics phenotype differences between groups using the most differentiating metabolites (VIP > 1.0) (Fig. 1**)**. The heatmap also shows the correlation between metabolites and their changes among groups by clusters. A more noticeable difference between C19/A and the other groups was observed on the score plot of two principal components using PCA (principle component analysis) (Fig. 2A). Further exploration was made using multiclass PLS-DA (partial least squares discriminate analysis, mPLS-DA) to discover which specific metabolites reveal the most significant differences between these groups. Figure 2B is a score plot produced by mPLS-DA (Q2 = 0.544, p = 0.01) showing that the centroid of C19/A deviates from the centroids of the other two ARDS groups (H1N1/A, PNA/A) and the ICU-controls (CTL). This deviation was confirmed again via a pairwise comparison between two groups using PLS-DA, as shown in Additional file 1: Fig. S1. Performance statistics of all pairwise comparison models are summarized in Table 2 and show good model performance (*Q*2 > 0.6). The second group in Table 2 was used as a reference group for comparison. In addition, from the comparison between C19/A and C19/P, the model (*Q*2 = 0.62) suggests there is a metabolomic change accounting for disease severity, i.e., patients with COVID-19 ARDS vs. patients with COVID-19 pneumonia admitted to the hospital but not sick enough to be admitted to the ICU have significantly different metabolic patterns.

**Table 2:** The quality and performance of the PLS-DA model fitted to the complete data are summarized for comparing five study groups pairwise

		Group	Reference	Profile	*N* (S)	*N* (M)	*N* (C)	*R*^2^*X*	*R*^2^*Y*	*Q*^2^	Se	Sp	AUROC
*Metabolomic deviation of a patient group from non-ARDS ventilated ICU control*													
	H1N1/A		CTL	Complete	50	131	2	0.30	0.84	0.70	1.00	1.00	1.00
				Signature	50	46	3	0.49	0.92	0.78	1.00	1.00	1.00
				Biomarkers	50	2	2	1.00	0.80	0.78	1.00	1.00	1.00
	PNA/A		CTL	Complete	50	131	2	0.27	0.77	0.63	1.00	0.96	0.99
				Signature	50	43	3	0.46	0.79	0.71	0.96	0.96	0.99
				Biomarkers	50	8	1	0.66	0.53	0.51	0.88	1.00	0.97
	C19/A		CTL	Complete	50	131	2	0.22	0.96	0.85	1.00	1.00	1.00
				Signature	50	47	2	0.41	0.95	0.92	1.00	1.00	1.00
				Biomarkers	50	4	1	0.71	0.82	0.81	1.00	1.00	1.00
*Metabolomic difference of ARDS patient groups*													
	PNA/A		H1N1/A	Complete	50	131	4	0.43	0.98	0.77	1.00	1.00	1.00
				Signature	50	41	3	0.53	0.95	0.87	1.00	1.00	1.00
				Biomarkers	50	3	2	0.97	0.66	0.62	0.92	0.92	0.98
	C19/A		H1N1/A	Complete	50	131	3	0.40	0.90	0.74	1.00	1.00	1.00
				Signature	50	45	3	0.54	0.90	0.79	1.00	1.00	1.00
				Biomarkers	50	7	1	0.61	0.67	0.66	1.00	1.00	1.00
	C19/A		PNA/A	Complete	50	131	2	0.33	0.92	0.82	1.00	1.00	1.00
				Signature	50	41	2	0.50	0.94	0.90	1.00	1.00	1.00
				Biomarkers	50	11	2	0.76	0.89	0.87	1.00	1.00	1.00
*Metabolomic comparison between ARDS ICU and non-ICU patients*													
	C19/A		C19/P	Complete	50	131	3	0.35	0.86	0.62	1.00	1.00	1.00
				Signature	50	36	3	0.47	0.83	0.66	1.00	1.00	1.00
				Biomarkers	50	1	1	1.00	0.30	0.28	0.72	0.72	0.81

Three different profiles are displayed: (1) *Complete* = profile consisting of all metabolites, (2) *Signature* = profile consisting of metabolites in which the score of variable importance on projection is greater than 1, and (3) *Biomarkers* = metabolites found by our selection method over 1,000 bootstrap samples with favorable metric to highlight differences between cohorts

CTL: non-ARDS ventilated control patients admitted to ICU, H1N1/A = H1N1-induced ARDS patients admitted to ICU, PNA/A = bacterial pneumonia-induced ARDS patients admitted to ICU, C19/A = SARS-CoV-2-induced ARDS patients admitted to ICU, C19/P = SARS-CoV-2 patients admitted to hospital but not sick enough to be admitted to ICU

N(S) = number of samples, N(M) = number of metabolites, N(C) = number of components

### Metabolomic signatures

This study defines a metabolomic signature as a set of metabolites with VIP > 1.0, differentiating four groups simultaneously. Note that the scores in Additional file 1: Table S1 are computed from mPLS-DA, and thirty-eight elements of the metabolomic signature are highlighted in grey. The same analogy is applied to the pairwise comparison, and PLS-DA computes the scores. Additional file 1: Table S2 listed 46, 43, and 47 metabolites differentiating H1N1/A, PNA/A, and C19/A from CTL, respectively. Similarly, Additional file 1: Table S3 listed 41, 45, and 41 metabolites showing differences between PNA/A and H1N1/A, C19/A and H1N1/A, and C19/A and PNA/A, respectively. Finally, the comparison between C19/A and C19/P can be differentiated using 36 metabolites, as shown in Additional file 1: Table S4.

We determined unique metabolomics signatures comprised of shared and specific metabolites among groups. The specific metabolites of each C19/A, H1N1/A, and PNA/A group were extracted using the nonparametric Wilcoxon method by pairwise comparison (Additional file 1: Table S6). Specific metabolites of each group were significantly (*p* < 0.05) changed compared to each other for ARDS cohorts or ARDS cohorts and the CTR cohort. Table 3 shows that the changes of C3OH, αaminoadipic acid, fumaric acid, lactate, and pyruvate were specific to C19/A compared to other groups, while these metabolites were not different among H1N/A, PNA, and CTL. In the same way, C16:1 and C:9 were specific to the H1N1/A cohort, while C12 DC, C14:2 OH, Lyso PC a C18:1, PC ae C36:0, and SM (OH)C22:1 were specific to the PNA/A cohort. These metabolites may play the role of biomarkers to differentiate ARDS patients with different causes, including COVID-19, H1N1 influenza, and bacterial pneumonia causes of ARDS.

**Table 3:** The table shows the specific metabolites that significantly changed in the related cohorts compared to the other ARDS and control cohorts

	Specific to C19/A	Specific to H1N1/A	Specific to PNA/A
C12 DC			**
C14:2 OH			**
C16:1		**	
C3OH	**		
C9		**	
Fumaric acid	**		
Indole acetic acid	*		
Lactic acid	*		
LysoPC a C18:1			**
PC ae C36:0			*
Pyruvic acid	**		
SM (OH) C24:1	*		
SM C16:1	*		
SM(OH) C22:1			**
SM(OH) C24:1	*		
α-Aminoadipic acid	**		

^*^Metabolite significantly changed (*p-*value < 0.05) only in the cohort compared to other ARDS cohorts. **Metabolite significantly changed (p-value < 0.05) in the cohort compared to other ARDS cohorts and the control cohort

### Metabolomic biomarker candidates

Metabolomic biomarker candidates were found using our selection rules (described in the Materials and Methods section) from 1,000 resamples. The proportion of captured metabolites are reported in Additional file 1: Tables S2a (deviance of H1N1/A, PNA/A, C19/A from CTL), S3a (difference between PNA/A and H1N1/A, between C19/A and H1N1/A, and between C19/A and PNA/A), and S4a (difference between C19/A and C19/P), respectively. The performance of PLS-DA models with the selected markers is summarized in Table 2.

**Fig. 3:**
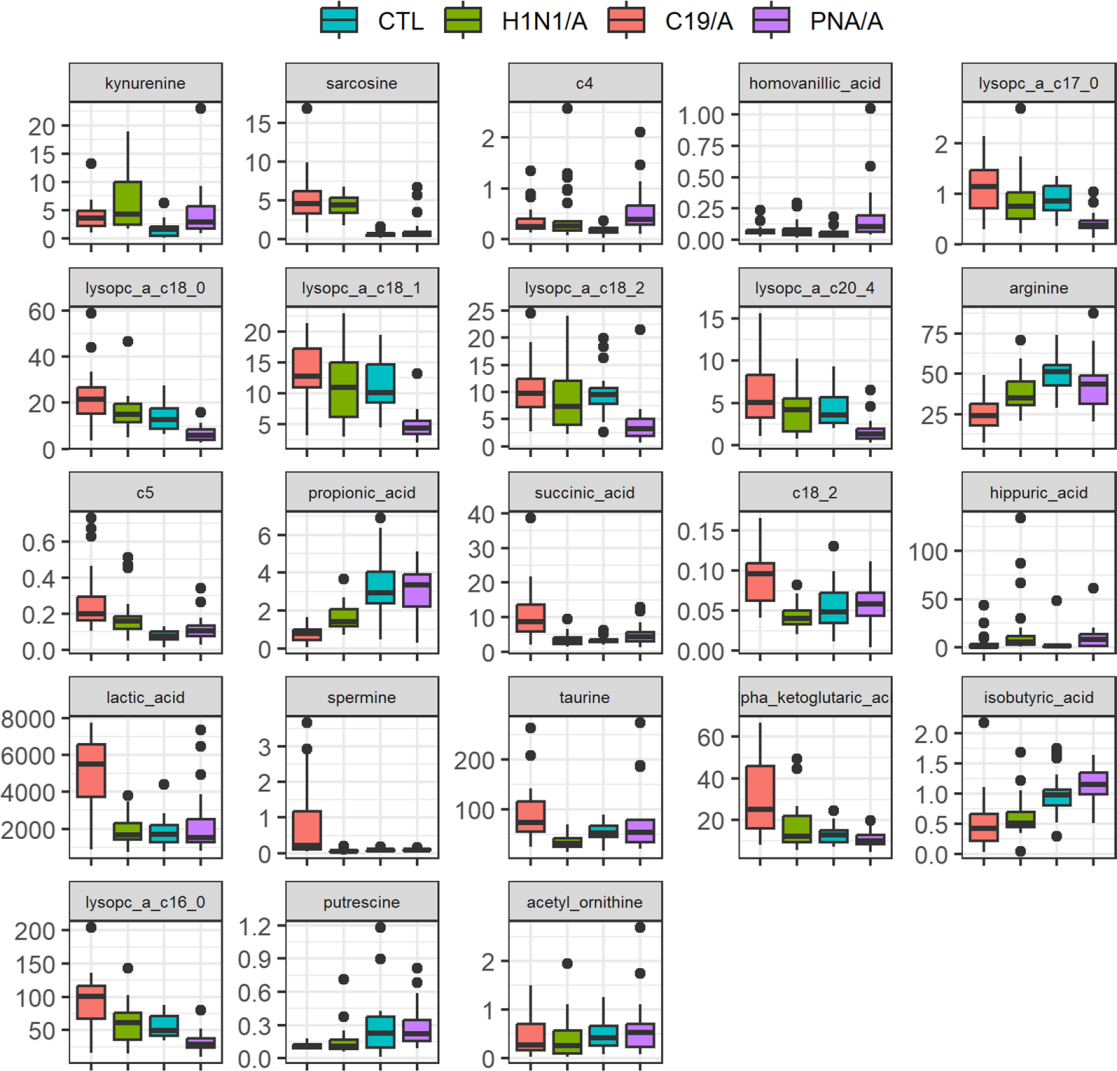
Side-by-side boxplot of biomarker quantification over study groups. CTL = non-ARDS ventilated control patients admitted to ICU, H1N1/A = H1N1-induced ARDS patients admitted to ICU, PNA/A = bacterial pneumonia-induced ARDS patients admitted to ICU, C19/A = COVID-19-induced ARDS patients admitted to ICU (C19/A)

For the models discussing the deviation from the control (i.e., ARDS groups vs ICU controls), we observed that both kynurenine and sarcosine concentration levels in H1N1/A are higher than CTL (Fig. 3). We also observed butyryl carnitine (c4), homovanillic acid, and kynurenine in PNA/A have higher concentration levels than CTL, while a family of lysoPC a C17:0, lysoPC a C18:0, lysoPC a C18:1, lysoPC a C18:2, lysoPC a C20:4 in PNA/A are lower than those in CTL. For the C19/A group, the concentration levels increased in acetylcarnitine (c5) and sarcosine compared to CTL, while the levels decreased in arginine and propionic acid compared to CTL.

From the models discussing two groups with different etiology, we observed that the concentration levels of lysoPC a C18:0, lysoPC a C18:1, and sarcosine in PNA/A are lower than H1N1/A. We also observed that C18:1, C18:2, lactic acid, spermine, succinic acid, and taurine are higher in C19/A than in H1N1/A, while the level of propionic acid is lower than H1N1/A. For the comparison between C19/A and PNA/A, the concentration levels increase in alpha-ketoglutaric acid, acetylcarnitine (c5), lysoPC a C16:0, lysoPC a C17:0, lysoPC a C18:0, lysoPC a C18:1, lysoPC a C20:4, and sarcosine in C19/A compared to PNA/A. In contrast, the levels of isobutyric acid, propionic acid, and putrescine decrease in C19/A vs. PNA/A. In addition, we observe that acetyl ornithine in C19/A is lower than in C19/P. Figure 3 shows side-by-side boxplots for the quantified biomarkers noted above.

### Performance evaluation

Model quality and performance of three metabolomic profiles (complete, signature, and biomarkers) are estimated and summarized in Table 2. We confirm that many metabolites are redundant from all pairwise comparisons since *R*^2^*X* (the proportion of variability explained by a model) increases as a profile has fewer metabolites, while *Q*^2^*Y* does not drop more than 0.1. Also, sensitivity and specificity are maintained over 0.9, even though fewer metabolites are included except for comparing C19/A and C19/P.

### A validation cohort of COVID-19 ARDS patients (C19/AV) is not different from C19/A patients when comparing metabolites in each group

To validate our COVID-19 ARDS (C19/A) metabolomic findings, we first attempted to discuss how different C19/A is from C19/AV (a validation cohort of 25 patients with COVID-19 ARDS admitted to the ICU). The means and standard deviations of these two groups are summarized in Additional file 1: Table S5. Based on the P-value adjusted by the Benjamini & Hochberg (BH) method, we found that all 131 metabolites except propionic acid were not significantly different at 0.05. We thus believed that C19/AV has reasonably similar characteristics to C19/A in terms of the metabolites used in this study. Subsequently, we performed a parallel comparison of C19/A and C19/AV to C19/A, H1N1, and PNA/A, using three criteria (adjusted p-value, fold-change/FC, and VIP). A decision for significance was discussed at 0.05 for adjusted p-value, 2 for FC, and 1 for VIP. We found that approximately 75.8% (adjusted p-value), 85.7% (FC), and 79.6% (VIP) of metabolites, respectively, in C19/A to C19/AV showed the same conclusion across the comparisons to C19/A, H1N1, and PNA/A.

### Pathway analysis

**Fig. 4:**
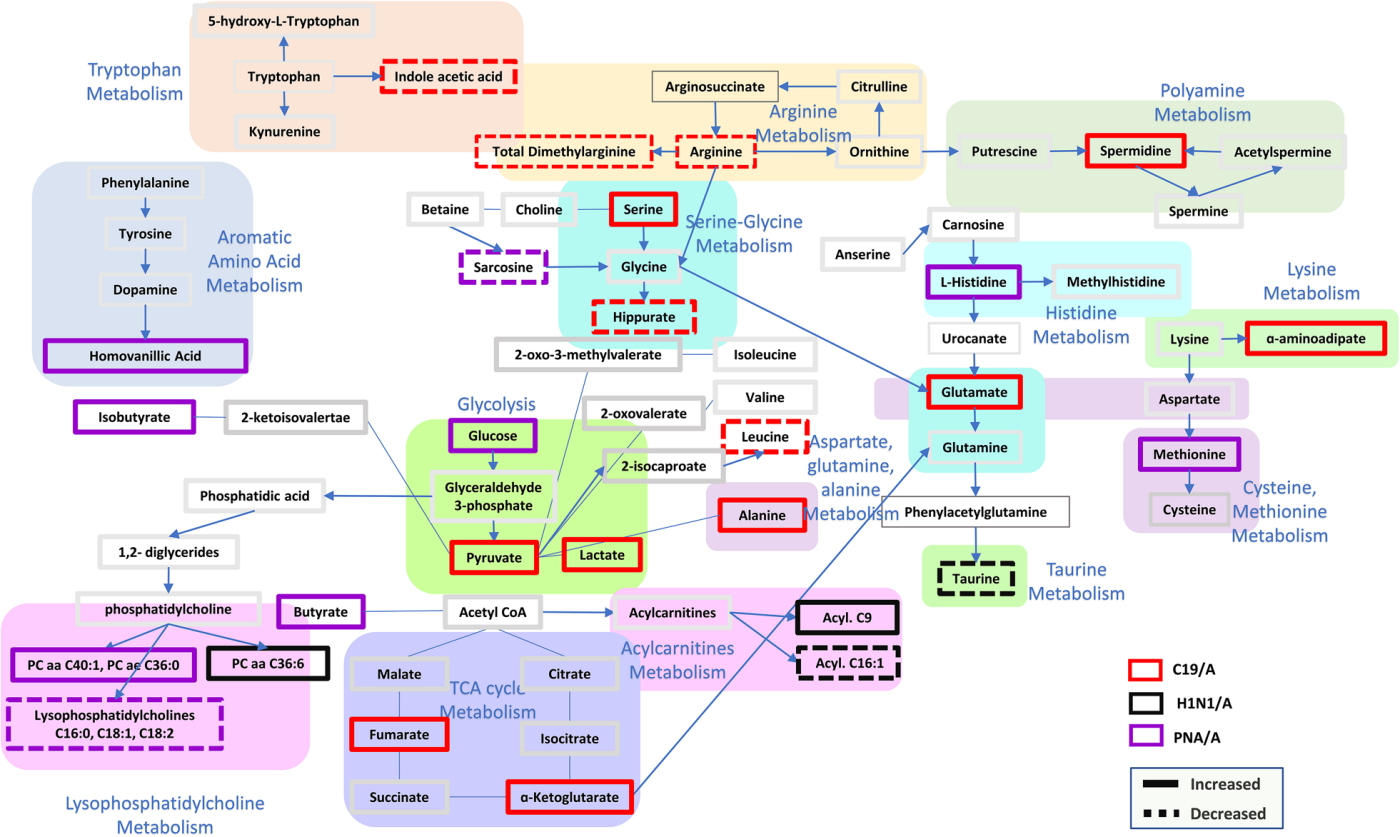
Metabolite and pathway illustration. Pathways included are those affected according to specific plasma metabolite changes in C19/A, H1N1/A, and PNA/A infections. Specific metabolites are included if they significantly changed in the group of interest compared to CTL, while they were not significantly different among the rest of the groups. The illustration of pathways is presented using KEGG pathway model descriptions

The metabolomic pathway differences are shown in Table 4**.** We have illustrated the metabolites and metabolic pathways involved in Fig. 4. For example, when examining metabolite-driven pathway analysis in viral causes of ARDS (between C19/A and H1N1/A), taurine and hypotaurine metabolism, pyruvate metabolism, citrate cycle (TCA cycle), lysine degradation and glycerophospholipid metabolism are involved. Comparing C19/A vs. PNA/A, differences were noted in D-glutamine and D-glutamate metabolism, arginine and proline metabolism, arginine biosynthesis, histidine metabolism, and pyruvate metabolism. Figure 4 illustrates the main affected biological pathway using the metabolites involved in each group and the KEGG pathway model descriptions. The higher number of specified metabolites in COVID-19 infection suggests more metabolic perturbation caused by the disease acuity in C19/A compared to H1N1/A and PNA/A. Arginine metabolism, aspartate, glutamine and alanine metabolism, pyruvate/lactate metabolism, TCA cycle, and polyamine metabolism were related to C19-specified metabolite changes. H1N1/A was characterized by taurine and acylcarnitine metabolisms. Also, homovanillic acid, methionine, PCs, and lysoPCs metabolisms were associated with PNA/A-specific metabolite changes.

**Table 4:** Pathway analysis from MetaboAnalyst 5.0 (https://www.metaboanalyst.ca/), including the most significant pathways

	Comparison	Pathway	log10(p)	P adj	FDR	Impact
Deviance from CTL	H1N1/A vs. CTL	Phenylalanine, tyrosine, and tryptophan biosynthesis	4.706	3.938E−04	8.204E−05	0.500
		Taurine and hypotaurine metabolism	4.480	6.289E−04	1.182E−04	0.429
		Phenylalanine metabolism	6.873	2.948E−06	8.374E−07	0.357
		Tryptophan metabolism	8.069	2.046E−07	1.066E−07	0.342
		Glycine, serine, and threonine metabolism	14.155	1.749E−13	1.749E−13	0.339
	PNA/A vs CTL	Phenylalanine, tyrosine, and tryptophan biosynthesis	7.432	8.883E−07	4.442E−07	0.500
		Phenylalanine metabolism	7.432	8.883E−07	4.442E−07	0.357
		Glycine, serine, and threonine metabolism	2.186	8.471E−02	1.303E−02	0.246
		Alanine, aspartate and glutamate metabolism	2.577	4.502E−02	7.038E−03	0.224
		Tryptophan metabolism	6.323	1.045E−05	3.802E−06	0.199
	C19/A vs CTL	Phenylalanine, tyrosine, and tryptophan biosynthesis	6.799	3.494E−06	3.857E−07	0.500
		Arginine and proline metabolism	10.693	6.086E−10	1.380E−10	0.372
		Arginine biosynthesis	12.028	2.906E−11	7.969E−12	0.365
		Phenylalanine metabolism	6.799	3.494E−06	3.857E−07	0.357
		Glycine, serine, and threonine metabolism	16.764	5.857E−16	5.857E−16	0.339
Comparison	PNA/A vs H1N1/A	Taurine and hypotaurine metabolism	3.346	8.119E−03	1.654E−03	0.429
		Arginine and proline metabolism	5.691	4.282E−05	2.243E−05	0.280
		Histidine metabolism	2.976	1.479E−02	2.324E−03	0.221
		Glycine, serine, and threonine metabolism	6.942	2.515E−06	2.515E−06	0.143
		Glycerophospholipid metabolism	3.968	2.151E−03	6.052E−04	0.138
	C19/A vs H1N1/A	Taurine and hypotaurine metabolism	6.748	4.172E−06	8.049E−07	0.429
		Pyruvate metabolism	6.497	6.368E−06	9.551E−07	0.207
		Citrate cycle (TCA cycle)	6.760	4.172E−06	8.049E−07	0.169
		Lysine degradation	2.875	9.325E−03	1.713E−03	0.141
		Glycerophospholipid metabolism	5.893	2.177E−05	3.065E−06	0.138
	C19/A vs PNA/A	D-glutamine and D-glutamate metabolism	2.963	8.707E−03	1.314E−03	0.500
		Arginine and proline metabolism	10.732	5.187E−10	2.686E−10	0.458
		Arginine biosynthesis	8.107	1.796E−07	3.141E−08	0.254
		Histidine metabolism	4.637	2.996E−04	3.932E−05	0.221
		Pyruvate metabolism	5.627	3.744E−05	4.566E−06	0.207
Severity	C19/A vs C19/P	Phenylalanine, tyrosine, and tryptophan biosynthesis	0.961	1.000E+00	1.314E−01	0.500
		Lysine degradation	2.877	3.054E−02	1.512E−02	0.141
		Tyrosine metabolism	0.961	1.000E+00	1.314E−01	0.140
		Glycerophospholipid metabolism	1.474	4.704E−01	7.331E−02	0.112
		Tryptophan metabolism	2.006	1.678E−01	2.960E−02	0.105

Metabolites used are only those whose score of variable importance on projection is greater than 1 in PLS-DA for comparing two groups between non-ARDS ventilated control patients admitted to ICU (CTL) and H1N1-induced ARDS admitted to ICU (H1N1/A), between CTL and bacterial pneumonia-induced ARDS patients admitted to ICU (PNA/A), between CTL and COVID-19-induced ARDS patients admitted to ICU (C19/A), between PNA/A and H1N1/A, between C19/A and H1N1/A, between C19/A and PNA/A, and between C19/A patients and COVID-19-induced non-ARDS patients admitted to hospital but not sick enough to be admitted to ICU (C19/P)

## Discussion

It has been proposed that ARDS due to COVID-19 has different clinical features compared with ARDS by other causes [25], including both viral-associated and bacterial pneumonia-associated ARDS [26]. Thus, we investigated whether different infectious causes of ARDS, both viral and bacterial, altered plasma metabolites reflecting different mechanisms of injury. Indeed, our study does show metabolomic differences between viral causes of ARDS (specifically, C19/A and H1N1/A) that were significant for taurine and hypotaurine metabolism, pyruvate metabolism, citrate cycle (TCA cycle), lysine degradation, and glycerophospholipid metabolism. We also found distinct differences between bacterial pneumonia-associated ARDS (PNA/A) and viral-associated ARDS (both C19/A and H1N1/A) in taurine and hypotaurine, arginine and proline, and histidine metabolisms. Finally, we found distinct metabolite differences in COVID-19 severity as reflected by those COVID-19 patients requiring ICU admissions (C19/A) vs those that do not (C19/P) in phenylalanine, tyrosine and tryptophan biosynthesis, lysine degradation, and tyrosine metabolism.

Our main finding is significant metabolomic differences between COVID-19 and other viral causes of ARDS, specifically H1N1. Metabolic pathways also differ between COVID-19 and bacterial pneumonia-associated ARDS and non-ARDS ICU-ventilated control patients. Our data also reveal a high level of similarity between the C19/A and C19/P involved metabolites when compared to H1N1/A, PNA/A, and CTL groups. COVID-19 ARDS is more severe than COVID-19 pneumonia without ARDS who are not admitted to the ICU and is characterized by increased branched-chain amino acids (BCAAs), glucose, some short- and long-chain acylcarnitines, and decreased acetyl-ornithine, propionic acid, and long phosphatidylcholines (PC 40:1 and 40:2).

We found that lipid metabolism is important in viral-mediated ARDS, as seen in the heatmap of COVID-19 ARDS and as noted by others for H1N1 [27] and COVID-19 [28]. This is an interesting and potentially important finding as there is early evidence that using a PCSK9 inhibitor alters COVID-19 inflammation and outcome [29]. Just how lipid metabolism affects inflammation in COVID-19 is not known, but there is an association between lipid disorders and COVID-19 severity [30], and several studies involving statin use in COVID-19 have been undertaken [30].

We also note that aromatic amino acid and lysine metabolism were highlighted in the differentiation of COVID-19 ARDS and COVID-19 pneumonia without ARDS. Our findings agree with others, where arginine metabolism, glycolytic pathway, and one-carbon metabolism were highlighted as the most perturbed metabolic phenotype in COVID-19 [31–33]. Importantly, this may be used to potentially predict those individuals with COVID-19 pneumonia that will develop more severe disease (i.e., ARDS) and, therefore, may need close attention for the need to transfer to the ICU. This may be a marker of COVID-19 severity; however, this will need to be validated in future investigations.

COVID-19 metabolomics studies have generally been compared to normal controls. There have been many small COVID-19 metabolomics studies; a few will be highlighted here. López-Hernández et al. [16] found differences in serum metabolites between COVID-19-negative and non-hospitalized COVID-19-positive individuals, including increased kynurenine/tryptophan ratio, lysoPC(aC26:0), and pyruvic acid. Examining COVID-19-positive non-hospitalized and hospitalized individuals, they found increased decanoylcarnitine (C10:2), butyric acid, and pyruvic acid. Finally, when COVID-19-positive hospitalized patients were compared to COVID-19-positive intubated patients, they found increased lysophosphatidylcholine (lysoPC aC28:0) [17]. When they compared severe COVID-19 patients to normal controls, they found increased glutamate, aspartic acid, kynurenine, and lysoPCs and decreased glutamine, citrulline, tryptophan, serotonin, and nicotinamide mononucleotide in the severe COVID-19 patients [18]. Examining plasma using targeted DI-MS/MS from three small groups: 10 patients with COVID-19, ten patients who were COVID-19 negative, and ten normal controls, they found kynurenine was the most significantly increased metabolite between COVID-19-positive and healthy controls and decreased metabolites included: arginine, sarcosine, lysoPC. They also found increased kynurenine and arginine/kynurenine ratio in COVID-19-positive vs. COVID-19-negative patients [34]. When serum and plasma profiles of COVID-19 patients were compared to healthy controls, they found involvement of tryptophan metabolism via the kynurenine pathway and elevated tryptophan, kynurenine, and 3-hydroxykynurenine. Blasco et al*.* [35] examined plasma in 55 COVID-19-positive patients and 45 healthy controls, and they found involvement of the cytosine and tryptophan‐nicotinamide pathways that were linked to the tryptophan-kynurenine pathway and increased cytosine levels in COVID-19 patients. Despite this wide range of metabolomics findings, the overall perturbed metabolic pathways currently observed in COVID-19 include pyruvate metabolism, kynurenine pathways, and amino acid metabolism; this was linked specifically to tryptophan metabolism [13–19]. Our findings, in general, agree with this summary. Our data revealed the same level of increased kynurenine in C19/A, H1N1/A, and PNA/A compared to CTL, suggesting immune dysregulation [36] due to viral and bacterial ARDS. Although it has been shown that the kynurenine/tryptophan ratio may be correlated with COVID-19 severity, our data showed non-significant kynurenine and tryptophan concentrations when C19/A and C19/P were compared. It has been shown that altered unsaturated lysophosphatidylcholines are associated with COVID-19 infection, with some lipid types showing decreased and others showing increased levels. Nonetheless, LysoPCs 16:0, 18:0, 18:1, and 18:2 were reduced in COVID-19-positive individuals [37, 38]. However, our findings showed these lysoPCs were significantly increased in C19/A and C19/P compared to H1N1/A, PNA/A, and CTL. Our data also demonstrated that LysoPCs C16:0, C18:1, and C18:2 were specifically elevated in PNA/A. The association of reduced LysoPC compounds with mortality and severity among bacterial CAP patients has been previously shown [39]. In a previous study, the metabolomic investigation of COVID-19 and H1N1 patients with ARDS showed distinct metabolic phenotypes between these two viral causes (with model characteristics showing *Q*2 = 0.89 and AUC = 1.0) [40]. Our data agreed with this study to show significantly increased glucose, lactate, glutamate, and fatty acid levels in COVID-19 ARDS vs. H1N1 ARDS. Data in the present study revealed specific metabolites involved with PNA/A that are significantly different from C19/A and H1N1/A; however, these were not significantly different between C19/A and H1N/A, suggesting that these metabolites could be specific to viral infections with ARDS compared with bacterial pneumonia. This suggests increased sarcosine, lysoPC 16:0, C18:1, C18:2, and decreased levels of homovanillic acid, isobutyrate, glucose, histidine, and methionine sulfoxide were associated with viral infections.

Just as we show the importance of different pathways between COVID-19 and other causes of ARDS, others have shown that aromatic amino acids and one-carbon metabolism differ between ARDS patients compared to healthy controls[8]. We extend these findings by showing that specific metabolomic pathways characterize different infectious causes of ARDS. COVID-19 ARDS had prominent arginine metabolism, H1N1 ARDS had increased taurine and hypotaurine metabolism, while bacterial pneumonia ARDS had increased alanine, aspartate, and glutamate metabolism.

COVID-19 ARDS is further differentiated by pyruvate metabolism and glutamine/glutamate metabolism compared to H1N1 ARDS and bacterial pneumonia ARDS. Notably, taurine/hypotaurine, histidine, and one-carbon metabolism were more specific to H1N1 ARDS.

Similarly, a previous ^1^H-NMR plasma metabolomics study examining H1N1 pneumonia vs. controls [27] shows many similar elevated metabolites (including beta-alanine, phenylalanine, and ornithine) and decreased (citrate, taurine, glycine, glutamine, and serine) metabolites as we show here using DI/LC–MS/MS. As previously shown, the data here reveal that aminoacyl-tRNA biosynthesis is the most impactful metabolomic pathway comparing H1N1 ARDS patients vs. ICU-ventilated controls.

ARDS is clinically heterogeneous [41–43], and our study and others [8] highlight several potential metabolomic sub-phenotypes of COVID-19 and other viral causes of ARDS. We add new insights regarding metabolomic sub-phenotypes within COVID-19 that mark the severity of illness such that COVID-19 pneumonia non-ICU patient metabolites differ from metabolites found in COVID-19 ARDS ICU patients. ARDS has been previously subphenotypes into hyper- and hypo-inflammatory using cytokine analyses [41–43]; however, we did not examine cytokines in this study. Others have begun exploring some metabolomics differences between hyper- and hypo-inflammatory ARDS phenotypes [22, 44].

We believe C19/AV was useful to validate the findings from C19/A externally. For this conclusion, we discussed how similar C19/A and C19/AV are using adjusted p-values from multiple tests. Subsequently, we showed from a parallel analysis that approximately 80% of metabolites have the same conclusion between C19/A and C19/AV compared to C19/P, H1N1, and PNA/A, respectively, with different measures.

One must consider limitations of this study, such as, a relatively small sample size in each cohort and the use of targeted quantitative metabolomics that captured only 143 metabolites. Finally, our cohorts were drawn from sample collections at three different periods or dates. Although all samples were prepared similarly and frozen at −80 °C and management of ARDS over this period has not changed significantly, these factors may have affected the results. However, we believe our findings are robust and we show this by applying 1,000 resampling to our analysis.

This study is unique in that it compares three infectious causes of ARDS (COVID-19, H1N1, and bacterial pneumonia-associated ARDS) as well as comparing COVID-19 pneumonia patients not sick enough to be admitted to the ICU vs COVID-19 ARDS admitted to the ICU. We found distinct differences in metabolites between bacterial pneumonia-associated ARDS (PNA/A) and viral-associated ARDS (caused by COVID-19 (C19/A) and H1N1 influenza (H1N1/A)). Importantly, we also see differences between viral causes of ARDS, namely COVID-19 ARDS (C19/A) and H1N1 ARDS (H1N1/A). Finally, we found metabolomics differences between COVID-19 pneumonia non-ICU patients and COVID-19 ARDS ICU patients with metabolite changes reflecting the severity of the disease (which may be used to help define which COVID-19 pneumonia patients may require ICU care early in the progression to ARDS).

### Supplementary Information

**Additional file 1.** Tables S1, S2, S2a, S3, S3a, S4, S4a, S5, and S6 and Figure S1.
