# Supplementary material for: Using a targeted metabolomics approach to explore differences in ARDS associated with COVID-19 compared to ARDS caused by H1N1 influenza and bacterial pneumonia
Source: Crit Care. 2024 Feb 27;28:63. doi: 10.1186/s13054-024-04843-0 (PMC10900651; doi:10.1186/s13054-024-04843-0)
Supplement: Supplementary file 1 — Additional file 1. Tables S1, S2, S2a, S3, S3a, S4, S4a, S5, and S6 and Figure S1. [file 13054_2024_4843_MOESM1_ESM.docx]

**Supplement**

**Table S1.** A multi-class PLS-DA model calculated the importance of the variable on the projection (VIP) scores, and the Boruta algorithm computed the number of hits. * is the metabolite marked as included in the final decision from the Boruta algorithm.

|  | Name | VIP/PLSDA | Hits/Boruta |  | Name | VIP/PLSDA | Hits/Boruta |
| --- | --- | --- | --- | --- | --- | --- | --- |
| 1 | acetyl_ornithine | 0.56 | 0.00 | 67 | isobutyric_acid | 1.28* | 1.00* |
| 2 | alanine | 1.10* | 0.84* | 68 | isoleucine | 0.79 | 0.00 |
| 3 | alpha_aminoadipic_acid | 0.93 | 0.69* | 69 | kynurenine | 1.39* | 0.97* |
| 4 | alpha_ketoglutaric_acid | 1.18* | 0.88* | 70 | lactic_acid | 1.32* | 1.00* |
| 5 | arginine | 1.12* | 1.00* | 71 | leucine | 0.79 | 0.02 |
| 6 | asparagine | 0.80 | 0.00 | 72 | lysine | 0.91 | 0.09 |
| 7 | aspartic_acid | 0.67 | 0.00 | 73 | lysopc_a_c14_0 | 0.63 | 0.00 |
| 8 | asymmetric_dimethylarginine | 0.96 | 0.89* | 74 | lysopc_a_c16_0 | 1.41* | 1.00* |
| 9 | beta_hydroxybutyric_acid | 0.20 | 0.00 | 75 | lysopc_a_c16_1 | 1.01* | 0.04 |
| 0 | betaine | 0.73 | 0.00 | 76 | lysopc_a_c17_0 | 1.36* | 1.00* |
| 11 | butyric_acid | 0.93 | 0.73* | 77 | lysopc_a_c18_0 | 1.49* | 1.00* |
| 12 | c0 | 0.57 | 0.00 | 78 | lysopc_a_c18_1 | 1.51* | 1.00* |
| 13 | c10 | 0.80 | 0.00 | 79 | lysopc_a_c18_2 | 1.34* | 1.00* |
| 14 | c10_1 | 1.00 | 0.02 | 80 | lysopc_a_c20_3 | 0.81 | 0.18 |
| 15 | c10_2 | 1.07* | 0.00 | 81 | lysopc_a_c20_4 | 1.37* | 1.00* |
| 16 | c12 | 0.63 | 0.01 | 82 | lysopc_a_c24_0 | 1.13* | 0.56 |
| 17 | c12_1 | 0.35 | 0.00 | 83 | lysopc_a_c26_0 | 0.86 | 0.00 |
| 18 | c12dc | 1.30* | 0.97* | 84 | lysopc_a_c26_1 | 0.88 | 0.00 |
| 19 | c14 | 1.06* | 0.99* | 85 | lysopc_a_c28_0 | 0.99 | 0.42 |
| 20 | c14_1 | 1.19* | 0.95* | 86 | lysopc_a_c28_1 | 0.90 | 0.11 |
| 21 | c14_1oh | 0.70 | 0.00 | 87 | methionine | 0.69 | 0.02 |
| 22 | c14_2 | 0.70 | 0.11 | 88 | methionine_sulfoxide | 1.01* | 0.76* |
| 23 | c14_2oh | 1.36* | 0.88* | 89 | methylhistidine | 0.71 | 0.00 |
| 24 | c16 | 1.11* | 0.79* | 90 | methylmalonic_acid | 0.90 | 0.06 |
| 25 | c16_1 | 1.00 | 0.03 | 91 | ornithine | 1.30* | 1.00* |
| 26 | c16_1oh | 0.57 | 0.00 | 92 | pc_aa_c32_2 | 1.28* | 0.02 |
| 27 | c16_2 | 1.19* | 0.86* | 93 | pc_aa_c36_0 | 0.89 | 0.00 |
| 28 | c16_2oh | 0.80 | 0.01 | 94 | pc_aa_c36_6 | 0.97 | 0.03 |
| 29 | c16oh | 0.84 | 0.34 | 95 | pc_aa_c38_0 | 0.72 | 0.00 |
| 30 | c18 | 0.96 | 0.78* | 96 | pc_aa_c38_6 | 0.76 | 0.00 |
| 31 | c18_1 | 1.16* | 1.00* | 97 | pc_aa_c40_1 | 1.04* | 0.02 |
| 32 | c18_1oh | 0.47 | 0.00 | 98 | pc_aa_c40_2 | 0.80 | 0.00 |
| 33 | c18_2 | 1.06* | 1.00* | 99 | pc_aa_c40_6 | 0.86 | 0.00 |
| 34 | c2 | 0.63 | 0.00 | 100 | pc_ae_c36_0 | 1.08* | 0.74* |
| 35 | c3 | 0.98 | 0.71* | 101 | pc_ae_c40_6 | 0.99 | 0.42 |
| 36 | c3_1 | 0.73 | 0.00 | 102 | phenylalanine | 1.31* | 1.00* |
| 37 | c3oh | 0.99 | 0.02 | 103 | proline | 0.30 | 0.00 |
| 38 | c4 | 1.05* | 0.97* | 104 | propionic_acid | 1.56* | 1.00* |
| 39 | c4_1 | 1.13* | 0.04 | 105 | putrescine | 1.07* | 0.94* |
| 40 | c4oh | 0.62 | 0.00 | 106 | pyruvic_acid | 1.17* | 0.87* |
| 41 | c5 | 1.53* | 1.00* | 107 | sarcosine | 1.97* | 1.00* |
| 42 | c5_1 | 0.92 | 0.02 | 108 | serine | 0.87 | 0.46 |
| 43 | c5_1dc | 0.90 | 0.00 | 109 | serotonin | 1.10* | 0.51 |
| 44 | c5dc | 0.82 | 0.01 | 110 | sm_c16_0 | 1.10* | 0.07 |
| 45 | c5mdc | 1.07* | 0.04 | 111 | sm_c16_1 | 0.95 | 0.00 |
| 46 | c5oh | 0.93 | 0.00 | 112 | sm_c18_0 | 1.02* | 0.00 |
| 47 | c6 | 0.65 | 0.00 | 113 | sm_c18_1 | 0.82 | 0.00 |
| 48 | c6_1 | 0.79 | 0.26 | 114 | sm_c20_2 | 1.55* | 0.91* |
| 49 | c7dc | 0.95 | 0.38 | 115 | sm_oh__c14_1 | 0.64 | 0.01 |
| 50 | c8 | 0.92 | 0.37 | 116 | sm_oh__c16_1 | 0.64 | 0.00 |
| 51 | c9 | 1.59* | 1.00* | 117 | sm_oh__c22_1 | 1.01* | 0.00 |
| 52 | choline | 1.20* | 0.69* | 118 | sm_oh__c22_2 | 0.67 | 0.00 |
| 53 | citric_acid | 0.58 | 0.98* | 119 | sm_oh__c24_1 | 0.79 | 0.00 |
| 54 | citrulline | 0.69 | 0.00 | 120 | spermidine | 1.07* | 0.83* |
| 55 | creatine | 0.44 | 0.00 | 121 | spermine | 1.42* | 1.00* |
| 56 | creatinine | 0.82 | 0.00 | 122 | succinic_acid | 1.37* | 1.00* |
| 57 | fumaric_acid | 0.72 | 0.04 | 123 | taurine | 1.39* | 1.00* |
| 58 | glucose | 0.74 | 1.00* | 124 | threonine | 0.77 | 0.54 |
| 59 | glutamic_acid | 0.80 | 0.00 | 125 | total_dimethylarginine | 0.99 | 0.07 |
| 60 | glutamine | 0.77 | 0.52 | 126 | trans_hydroxyproline | 1.17* | 0.46 |
| 61 | glycine | 0.77 | 0.35 | 127 | trimethylamine_n_oxide | 0.55 | 0.00 |
| 62 | hippuric_acid | 1.17* | 0.97* | 128 | tryptophan | 0.70 | 0.00 |
| 63 | histidine | 1.10* | 0.45 | 129 | tyrosine | 0.59 | 0.00 |
| 64 | homocysteine | 0.45 | 0.00 | 130 | uric_acid | 0.87 | 0.03 |
| 65 | homovanillic_acid | 1.23* | 0.47 | 131 | valine | 0.90 | 0.00 |
| 66 | indole_acetic_acid | 1.04* | 0.35 |  |  |  |  |

**Table S2.** Variable importance on the projection (VIP) scores calculated by PLS-DA separating between CTL (non-ARDS ventilated control patients admitted to ICU) and H1N1/A (H1N1-induced ARDS patients admitted to ICU), between CTL and PNA/A (bacterial pneumonia-induced ARDS patients admitted to ICU), and between CTL and C19/A (SARS-CoV-2 induced ARDS patients admitted to ICU). * is a biomarker selected by our selection method over 1,000 bootstrap samples.

|  | Name | H1N1/A | PNA/A | C19/A |  | Name | H1N1/A | PNA/A | C19/A |
| --- | --- | --- | --- | --- | --- | --- | --- | --- | --- |
| 1 | acetyl_ornithine | 0.70 | 0.16 | 0.47 | 67 | isobutyric_acid | 1.22 | 0.40 | 1.67 |
| 2 | alanine | 1.31 | 0.69 | 0.55 | 68 | isoleucine | 0.94 | 0.21 | 1.17 |
| 3 | alpha_aminoadipic_acid | 0.26 | 0.28 | 1.19 | 69 | kynurenine | 2.43* | 1.79* | 1.76 |
| 4 | alpha_ketoglutaric_acid | 0.30 | 0.42 | 1.50 | 70 | lactic_acid | 0.17 | 0.30 | 1.86 |
| 5 | arginine | 0.83 | 0.70 | 1.81* | 71 | leucine | 0.34 | 0.54 | 1.24 |
| 6 | asparagine | 0.11 | 1.20 | 0.25 | 72 | lysine | 1.32 | 0.78 | 1.06 |
| 7 | aspartic_acid | 0.62 | 1.03 | 1.00 | 73 | lysopc_a_c14_0 | 0.88 | 0.31 | 0.67 |
| 8 | asymmetric_dimethylarginine | 0.44 | 0.65 | 1.17 | 74 | lysopc_a_c16_0 | 0.75 | 1.54 | 0.90 |
| 9 | beta_hydroxybutyric_acid | 0.22 | 0.40 | 0.42 | 75 | lysopc_a_c16_1 | 1.11 | 1.73 | 0.90 |
| 0 | betaine | 0.41 | 0.86 | 0.21 | 76 | lysopc_a_c17_0 | 0.70 | 1.83* | 0.47 |
| 11 | butyric_acid | 0.46 | 0.77 | 0.95 | 77 | lysopc_a_c18_0 | 0.79 | 1.58* | 0.88 |
| 12 | c0 | 0.58 | 0.69 | 0.31 | 78 | lysopc_a_c18_1 | 0.69 | 2.06* | 0.33 |
| 13 | c10 | 1.30 | 0.72 | 0.38 | 79 | lysopc_a_c18_2 | 0.77 | 2.03* | 0.07 |
| 14 | c10_1 | 0.85 | 0.37 | 0.82 | 80 | lysopc_a_c20_3 | 0.85 | 0.33 | 0.49 |
| 15 | c10_2 | 1.46 | 0.16 | 0.36 | 81 | lysopc_a_c20_4 | 0.77 | 1.86 | 0.39 |
| 16 | c12 | 0.38 | 0.20 | 0.73 | 82 | lysopc_a_c24_0 | 0.91 | 1.85* | 1.23 |
| 17 | c12_1 | 0.38 | 0.08 | 0.23 | 83 | lysopc_a_c26_0 | 1.34 | 1.07 | 1.35 |
| 18 | c12dc | 1.09 | 0.86 | 0.59 | 84 | lysopc_a_c26_1 | 0.57 | 1.29 | 0.95 |
| 19 | c14 | 0.54 | 0.75 | 0.89 | 85 | lysopc_a_c28_0 | 0.74 | 0.83 | 1.61 |
| 20 | c14_1 | 1.90 | 0.58 | 0.92 | 86 | lysopc_a_c28_1 | 1.00 | 1.41 | 1.32 |
| 21 | c14_1oh | 0.71 | 0.30 | 0.84 | 87 | methionine | 0.51 | 1.19 | 0.35 |
| 22 | c14_2 | 1.11 | 1.04 | 0.47 | 88 | methionine_sulfoxide | 1.29 | 1.72 | 0.63 |
| 23 | c14_2oh | 1.28 | 0.74 | 0.19 | 89 | methylhistidine | 0.69 | 0.66 | 0.53 |
| 24 | c16 | 0.66 | 0.09 | 1.02 | 90 | methylmalonic_acid | 1.17 | 1.56 | 0.68 |
| 25 | c16_1 | 0.70 | 0.85 | 0.33 | 91 | ornithine | 1.44 | 0.32 | 1.92 |
| 26 | c16_1oh | 0.68 | 0.44 | 0.29 | 92 | pc_aa_c32_2 | 1.62 | 0.95 | 0.50 |
| 27 | c16_2 | 0.99 | 0.56 | 1.19 | 93 | pc_aa_c36_0 | 0.21 | 0.51 | 1.14 |
| 28 | c16_2oh | 0.94 | 1.47 | 0.71 | 94 | pc_aa_c36_6 | 0.70 | 1.13 | 1.42 |
| 29 | c16oh | 0.61 | 0.33 | 0.65 | 95 | pc_aa_c38_0 | 1.03 | 0.34 | 0.74 |
| 30 | c18 | 0.52 | 0.16 | 0.92 | 96 | pc_aa_c38_6 | 1.32 | 0.86 | 0.97 |
| 31 | c18_1 | 0.17 | 0.85 | 1.60 | 97 | pc_aa_c40_1 | 0.91 | 0.73 | 0.90 |
| 32 | c18_1oh | 0.40 | 0.36 | 0.47 | 98 | pc_aa_c40_2 | 1.06 | 1.20 | 0.68 |
| 33 | c18_2 | 0.40 | 0.29 | 1.17 | 99 | pc_aa_c40_6 | 1.35 | 0.60 | 1.06 |
| 34 | c2 | 1.03 | 1.08 | 0.86 | 100 | pc_ae_c36_0 | 0.37 | 1.64 | 0.31 |
| 35 | c3 | 0.72 | 1.33 | 1.24 | 101 | pc_ae_c40_6 | 1.50 | 0.91 | 1.48 |
| 36 | c3_1 | 1.07 | 0.74 | 0.20 | 102 | phenylalanine | 1.70 | 2.19 | 1.71 |
| 37 | c3oh | 0.47 | 0.21 | 1.27 | 103 | proline | 0.15 | 0.79 | 0.14 |
| 38 | c4 | 1.35 | 1.90* | 1.12 | 104 | propionic_acid | 1.12 | 0.43 | 2.43* |
| 39 | c4_1 | 1.51 | 0.65 | 0.36 | 105 | putrescine | 1.21 | 0.60 | 1.14 |
| 40 | c4oh | 1.06 | 1.13 | 0.85 | 106 | pyruvic_acid | 0.58 | 0.11 | 1.74 |
| 41 | c5 | 1.91 | 0.79 | 2.36* | 107 | sarcosine | 2.84* | 0.48 | 2.51* |
| 42 | c5_1 | 1.50 | 1.10 | 1.25 | 108 | serine | 0.77 | 1.45 | 0.60 |
| 43 | c5_1dc | 0.42 | 0.83 | 0.98 | 109 | serotonin | 1.64 | 1.57 | 0.50 |
| 44 | c5dc | 0.93 | 0.11 | 0.10 | 110 | sm_c16_0 | 1.10 | 1.68 | 0.48 |
| 45 | c5mdc | 0.74 | 0.76 | 0.70 | 111 | sm_c16_1 | 1.11 | 0.95 | 0.24 |
| 46 | c5oh | 0.89 | 0.77 | 0.74 | 112 | sm_c18_0 | 1.22 | 1.45 | 0.60 |
| 47 | c6 | 0.45 | 1.17 | 0.23 | 113 | sm_c18_1 | 0.99 | 0.87 | 0.09 |
| 48 | c6_1 | 0.73 | 0.62 | 0.51 | 114 | sm_c20_2 | 0.95 | 1.24 | 1.27 |
| 49 | c7dc | 1.18 | 1.17 | 0.39 | 115 | sm_oh__c14_1 | 0.41 | 0.73 | 0.42 |
| 50 | c8 | 1.40 | 1.57 | 0.92 | 116 | sm_oh__c16_1 | 0.68 | 0.88 | 0.32 |
| 51 | c9 | 2.26 | 0.84 | 0.23 | 117 | sm_oh__c22_1 | 0.90 | 1.15 | 0.49 |
| 52 | choline | 0.79 | 0.52 | 1.25 | 118 | sm_oh__c22_2 | 0.77 | 1.12 | 0.41 |
| 53 | citric_acid | 0.90 | 0.77 | 1.03 | 119 | sm_oh__c24_1 | 0.72 | 0.92 | 0.75 |
| 54 | citrulline | 0.64 | 0.63 | 1.14 | 120 | spermidine | 0.74 | 0.60 | 1.08 |
| 55 | creatine | 0.59 | 0.98 | 0.17 | 121 | spermine | 0.59 | 0.23 | 1.62 |
| 56 | creatinine | 0.93 | 0.80 | 0.31 | 122 | succinic_acid | 0.33 | 0.62 | 1.82 |
| 57 | fumaric_acid | 0.31 | 0.45 | 1.08 | 123 | taurine | 1.25 | 0.26 | 0.82 |
| 58 | glucose | 0.55 | 0.11 | 1.22 | 124 | threonine | 1.29 | 1.44 | 0.43 |
| 59 | glutamic_acid | 0.41 | 0.91 | 0.45 | 125 | total_dimethylarginine | 1.09 | 1.11 | 0.32 |
| 60 | glutamine | 0.64 | 0.61 | 1.16 | 126 | trans_hydroxyproline | 0.59 | 1.18 | 1.03 |
| 61 | glycine | 1.15 | 1.51 | 1.35 | 127 | trimethylamine_n_oxide | 0.34 | 0.86 | 0.31 |
| 62 | hippuric_acid | 1.44 | 0.99 | 0.34 | 128 | tryptophan | 1.17 | 0.55 | 0.77 |
| 63 | histidine | 1.31 | 0.67 | 1.19 | 129 | tyrosine | 0.52 | 0.70 | 0.94 |
| 64 | homocysteine | 0.62 | 0.59 | 0.70 | 130 | uric_acid | 0.74 | 0.30 | 0.49 |
| 65 | homovanillic_acid | 0.92 | 2.10* | 0.73 | 131 | valine | 0.40 | 0.61 | 0.99 |
| 66 | indole_acetic_acid | 0.69 | 0.67 | 1.39 |  |  |  |  |  |

**Table S2a**. Proportions of biomarkers were found by our selection method over 1,000 bootstrap samples. Partial least square, fold-change, adjusted p-value, and elastic-net penalized logistic regression models were employed for comparing two groups between CTL (non-ARDS ventilated control patients admitted to ICU ) and H1N1/A (H1N1-induced ARDS patients admitted to ICU), between CTL and PNA/A (bacterial pneumonia-induced ARDS patients admitted to ICU), and between CTL and C19/A (SARS-CoV-2 induced ARDS patients admitted to ICU).

|  | Name | H1N1/A | PNA/A | C19/A |  | Name | H1N1/A | PNA/A | C19/A |
| --- | --- | --- | --- | --- | --- | --- | --- | --- | --- |
| 1 | acetyl_ornithine | 0.08 | 0.00 | 0.03 | 67 | isobutyric_acid | 0.63 | 0.02 | 0.74 |
| 2 | alanine | 0.66 | 0.02 | 0.01 | 68 | isoleucine | 0.18 | 0.00 | 0.54 |
| 3 | alpha_aminoadipic_acid | 0.00 | 0.00 | 0.58 | 69 | kynurenine | 0.96 | 0.95 | 0.85 |
| 4 | alpha_ketoglutaric_acid | 0.00 | 0.08 | 0.85 | 70 | lactic_acid | 0.00 | 0.00 | 0.89 |
| 5 | arginine | 0.47 | 0.25 | 0.96 | 71 | leucine | 0.01 | 0.03 | 0.53 |
| 6 | asparagine | 0.00 | 0.55 | 0.00 | 72 | lysine | 0.60 | 0.03 | 0.56 |
| 7 | aspartic_acid | 0.08 | 0.43 | 0.43 | 73 | lysopc_a_c14_0 | 0.07 | 0.01 | 0.03 |
| 8 | asymmetric_dimethylarginine | 0.00 | 0.05 | 0.68 | 74 | lysopc_a_c16_0 | 0.00 | 0.74 | 0.46 |
| 9 | beta_hydroxybutyric_acid | 0.00 | 0.01 | 0.01 | 75 | lysopc_a_c16_1 | 0.29 | 0.68 | 0.33 |
| 0 | betaine | 0.01 | 0.22 | 0.00 | 76 | lysopc_a_c17_0 | 0.00 | 0.94 | 0.05 |
| 11 | butyric_acid | 0.03 | 0.33 | 0.08 | 77 | lysopc_a_c18_0 | 0.03 | 0.92 | 0.44 |
| 12 | c0 | 0.01 | 0.01 | 0.00 | 78 | lysopc_a_c18_1 | 0.00 | 1.00 | 0.03 |
| 13 | c10 | 0.50 | 0.08 | 0.01 | 79 | lysopc_a_c18_2 | 0.03 | 0.92 | 0.00 |
| 14 | c10_1 | 0.04 | 0.00 | 0.20 | 80 | lysopc_a_c20_3 | 0.13 | 0.01 | 0.04 |
| 15 | c10_2 | 0.53 | 0.00 | 0.00 | 81 | lysopc_a_c20_4 | 0.00 | 0.98 | 0.04 |
| 16 | c12 | 0.00 | 0.01 | 0.06 | 82 | lysopc_a_c24_0 | 0.15 | 0.75 | 0.58 |
| 17 | c12_1 | 0.00 | 0.00 | 0.01 | 83 | lysopc_a_c26_0 | 0.62 | 0.60 | 0.67 |
| 18 | c12dc | 0.40 | 0.47 | 0.07 | 84 | lysopc_a_c26_1 | 0.00 | 0.56 | 0.29 |
| 19 | c14 | 0.06 | 0.21 | 0.36 | 85 | lysopc_a_c28_0 | 0.11 | 0.24 | 0.73 |
| 20 | c14_1 | 0.77 | 0.10 | 0.36 | 86 | lysopc_a_c28_1 | 0.26 | 0.60 | 0.65 |
| 21 | c14_1oh | 0.05 | 0.00 | 0.23 | 87 | methionine | 0.02 | 0.43 | 0.02 |
| 22 | c14_2 | 0.55 | 0.20 | 0.07 | 88 | methionine_sulfoxide | 0.64 | 0.79 | 0.07 |
| 23 | c14_2oh | 0.57 | 0.18 | 0.00 | 89 | methylhistidine | 0.01 | 0.01 | 0.05 |
| 24 | c16 | 0.13 | 0.00 | 0.51 | 90 | methylmalonic_acid | 0.40 | 0.69 | 0.08 |
| 25 | c16_1 | 0.13 | 0.09 | 0.00 | 91 | ornithine | 0.66 | 0.01 | 0.80 |
| 26 | c16_1oh | 0.00 | 0.02 | 0.00 | 92 | pc_aa_c32_2 | 0.50 | 0.03 | 0.00 |
| 27 | c16_2 | 0.33 | 0.06 | 0.58 | 93 | pc_aa_c36_0 | 0.00 | 0.04 | 0.50 |
| 28 | c16_2oh | 0.16 | 0.50 | 0.04 | 94 | pc_aa_c36_6 | 0.02 | 0.46 | 0.53 |
| 29 | c16oh | 0.04 | 0.01 | 0.10 | 95 | pc_aa_c38_0 | 0.19 | 0.01 | 0.18 |
| 30 | c18 | 0.05 | 0.01 | 0.38 | 96 | pc_aa_c38_6 | 0.59 | 0.23 | 0.41 |
| 31 | c18_1 | 0.00 | 0.11 | 0.73 | 97 | pc_aa_c40_1 | 0.12 | 0.01 | 0.26 |
| 32 | c18_1oh | 0.00 | 0.01 | 0.01 | 98 | pc_aa_c40_2 | 0.18 | 0.16 | 0.01 |
| 33 | c18_2 | 0.03 | 0.01 | 0.57 | 99 | pc_aa_c40_6 | 0.60 | 0.03 | 0.50 |
| 34 | c2 | 0.14 | 0.15 | 0.07 | 100 | pc_ae_c36_0 | 0.02 | 0.78 | 0.00 |
| 35 | c3 | 0.02 | 0.73 | 0.59 | 101 | pc_ae_c40_6 | 0.62 | 0.24 | 0.65 |
| 36 | c3_1 | 0.20 | 0.07 | 0.00 | 102 | phenylalanine | 0.67 | 0.81 | 0.69 |
| 37 | c3oh | 0.00 | 0.00 | 0.55 | 103 | proline | 0.00 | 0.03 | 0.00 |
| 38 | c4 | 0.68 | 0.99 | 0.68 | 104 | propionic_acid | 0.71 | 0.01 | 1.00 |
| 39 | c4_1 | 0.53 | 0.07 | 0.00 | 105 | putrescine | 0.29 | 0.01 | 0.61 |
| 40 | c4oh | 0.23 | 0.22 | 0.09 | 106 | pyruvic_acid | 0.08 | 0.00 | 0.82 |
| 41 | c5 | 0.88 | 0.19 | 1.00 | 107 | sarcosine | 1.00 | 0.13 | 1.00 |
| 42 | c5_1 | 0.62 | 0.51 | 0.57 | 108 | serine | 0.15 | 0.64 | 0.00 |
| 43 | c5_1dc | 0.00 | 0.12 | 0.28 | 109 | serotonin | 0.84 | 0.84 | 0.03 |
| 44 | c5dc | 0.09 | 0.00 | 0.00 | 110 | sm_c16_0 | 0.23 | 0.67 | 0.00 |
| 45 | c5mdc | 0.01 | 0.19 | 0.11 | 111 | sm_c16_1 | 0.21 | 0.14 | 0.00 |
| 46 | c5oh | 0.06 | 0.10 | 0.04 | 112 | sm_c18_0 | 0.29 | 0.29 | 0.04 |
| 47 | c6 | 0.00 | 0.23 | 0.00 | 113 | sm_c18_1 | 0.12 | 0.03 | 0.00 |
| 48 | c6_1 | 0.08 | 0.01 | 0.03 | 114 | sm_c20_2 | 0.13 | 0.53 | 0.58 |
| 49 | c7dc | 0.42 | 0.38 | 0.01 | 115 | sm_oh__c14_1 | 0.02 | 0.03 | 0.01 |
| 50 | c8 | 0.49 | 0.73 | 0.21 | 116 | sm_oh__c16_1 | 0.04 | 0.04 | 0.00 |
| 51 | c9 | 0.81 | 0.13 | 0.02 | 117 | sm_oh__c22_1 | 0.06 | 0.35 | 0.03 |
| 52 | choline | 0.10 | 0.04 | 0.52 | 118 | sm_oh__c22_2 | 0.03 | 0.20 | 0.01 |
| 53 | citric_acid | 0.64 | 0.22 | 0.27 | 119 | sm_oh__c24_1 | 0.02 | 0.03 | 0.14 |
| 54 | citrulline | 0.06 | 0.06 | 0.52 | 120 | spermidine | 0.37 | 0.55 | 0.59 |
| 55 | creatine | 0.04 | 0.23 | 0.00 | 121 | spermine | 0.36 | 0.01 | 0.84 |
| 56 | creatinine | 0.05 | 0.04 | 0.01 | 122 | succinic_acid | 0.00 | 0.20 | 0.94 |
| 57 | fumaric_acid | 0.00 | 0.03 | 0.48 | 123 | taurine | 0.68 | 0.00 | 0.31 |
| 58 | glucose | 0.48 | 0.01 | 0.53 | 124 | threonine | 0.56 | 0.49 | 0.02 |
| 59 | glutamic_acid | 0.01 | 0.32 | 0.03 | 125 | total_dimethylarginine | 0.25 | 0.43 | 0.02 |
| 60 | glutamine | 0.08 | 0.02 | 0.58 | 126 | trans_hydroxyproline | 0.17 | 0.37 | 0.48 |
| 61 | glycine | 0.58 | 0.49 | 0.44 | 127 | trimethylamine_n_oxide | 0.01 | 0.21 | 0.05 |
| 62 | hippuric_acid | 0.89 | 0.66 | 0.07 | 128 | tryptophan | 0.40 | 0.03 | 0.10 |
| 63 | histidine | 0.58 | 0.02 | 0.58 | 129 | tyrosine | 0.02 | 0.08 | 0.31 |
| 64 | homocysteine | 0.03 | 0.06 | 0.10 | 130 | uric_acid | 0.04 | 0.01 | 0.01 |
| 65 | homovanillic_acid | 0.14 | 0.91 | 0.24 | 131 | valine | 0.01 | 0.02 | 0.40 |
| 66 | indole_acetic_acid | 0.04 | 0.01 | 0.72 |  |  |  |  |  |

**Table S3.** Variable importance on the projection (VIP) scores calculated by PLS-DA separating two groups between bacterial pneumonia-induced ARDS patients admitted to ICU (PNA/A) and H1N1-induced ARDS patients admitted to ICU (H1N1/A), between SARS-CoV-2 induced ARDS patients admitted to ICU (C19/A) and H1N1/A, and between C19/A and PNA/A. * is a biomarker found by our selection method over 1,000 bootstrap samples.

|  | Name | PNA/A vs H1N1/A | C19/A vs H1N1/A | C19/A vs PNA/A |  | Name | PNA/A vs H1N1/A | C19/A vs H1N1/A | C19/A vs PNA/A |
| --- | --- | --- | --- | --- | --- | --- | --- | --- | --- |
| 1 | acetyl_ornithine | 0.80 | 0.19 | 0.49 | 67 | isobutyric_acid | 1.46 | 0.74 | 1.76* |
| 2 | alanine | 0.99 | 1.47 | 0.59 | 68 | isoleucine | 0.75 | 0.48 | 0.90 |
| 3 | alpha_aminoadipic_acid | 0.23 | 1.27 | 1.17 | 69 | kynurenine | 0.84 | 0.56 | 0.54 |
| 4 | alpha_ketoglutaric_acid | 0.74 | 1.28 | 1.73* | 70 | lactic_acid | 0.17 | 1.76* | 1.46 |
| 5 | arginine | 0.42 | 1.16 | 1.27 | 71 | leucine | 0.58 | 0.98 | 0.69 |
| 6 | asparagine | 1.26 | 0.46 | 0.95 | 72 | lysine | 1.28 | 0.40 | 0.80 |
| 7 | aspartic_acid | 0.76 | 0.61 | 0.50 | 73 | lysopc_a_c14_0 | 0.65 | 0.46 | 0.48 |
| 8 | asymmetric_dimethylarginine | 0.80 | 1.38 | 1.42 | 74 | lysopc_a_c16_0 | 1.68 | 1.03 | 2.13* |
| 9 | beta_hydroxybutyric_acid | 0.17 | 0.20 | 0.11 | 75 | lysopc_a_c16_1 | 1.03 | 0.53 | 0.79 |
| 0 | betaine | 1.14 | 0.27 | 0.77 | 76 | lysopc_a_c17_0 | 1.79 | 0.71 | 1.92* |
| 11 | butyric_acid | 1.21 | 1.04 | 1.41 | 77 | lysopc_a_c18_0 | 2.02* | 0.75 | 2.14* |
| 12 | c0 | 0.80 | 0.44 | 0.51 | 78 | lysopc_a_c18_1 | 2.05* | 0.66 | 2.05* |
| 13 | c10 | 0.66 | 0.92 | 0.57 | 79 | lysopc_a_c18_2 | 1.72 | 0.63 | 1.76 |
| 14 | c10_1 | 0.66 | 1.35 | 0.81 | 80 | lysopc_a_c20_3 | 1.24 | 0.39 | 0.73 |
| 15 | c10_2 | 1.30 | 1.12 | 0.60 | 81 | lysopc_a_c20_4 | 1.73 | 0.87 | 1.95* |
| 16 | c12 | 0.56 | 0.84 | 0.83 | 82 | lysopc_a_c24_0 | 1.22 | 0.57 | 0.50 |
| 17 | c12_1 | 0.39 | 0.54 | 0.30 | 83 | lysopc_a_c26_0 | 0.56 | 0.44 | 0.44 |
| 18 | c12dc | 2.05 | 0.57 | 1.19 | 84 | lysopc_a_c26_1 | 1.03 | 0.68 | 0.27 |
| 19 | c14 | 0.53 | 1.42 | 1.43 | 85 | lysopc_a_c28_0 | 0.34 | 1.04 | 0.79 |
| 20 | c14_1 | 1.46 | 0.89 | 0.59 | 86 | lysopc_a_c28_1 | 0.60 | 0.60 | 0.19 |
| 21 | c14_1oh | 0.92 | 0.63 | 0.88 | 87 | methionine | 1.01 | 0.36 | 0.68 |
| 22 | c14_2 | 0.71 | 0.56 | 0.51 | 88 | methionine_sulfoxide | 0.86 | 0.71 | 1.09 |
| 23 | c14_2oh | 2.11 | 1.10 | 0.84 | 89 | methylhistidine | 0.41 | 0.99 | 0.78 |
| 24 | c16 | 0.74 | 1.62 | 0.96 | 90 | methylmalonic_acid | 0.66 | 0.61 | 0.85 |
| 25 | c16_1 | 1.50 | 0.88 | 0.61 | 91 | ornithine | 1.25 | 0.71 | 1.56 |
| 26 | c16_1oh | 0.87 | 0.52 | 0.51 | 92 | pc_aa_c32_2 | 1.08 | 1.53 | 0.60 |
| 27 | c16_2 | 1.60 | 0.65 | 1.57 | 93 | pc_aa_c36_0 | 0.71 | 1.29 | 0.55 |
| 28 | c16_2oh | 0.50 | 0.30 | 0.58 | 94 | pc_aa_c36_6 | 0.80 | 1.09 | 0.34 |
| 29 | c16oh | 0.86 | 1.22 | 0.48 | 95 | pc_aa_c38_0 | 0.88 | 0.70 | 0.55 |
| 30 | c18 | 0.78 | 1.41 | 0.73 | 96 | pc_aa_c38_6 | 0.67 | 0.69 | 0.48 |
| 31 | c18_1 | 0.65 | 1.45* | 0.90 | 97 | pc_aa_c40_1 | 1.24 | 0.72 | 1.21 |
| 32 | c18_1oh | 0.65 | 0.59 | 0.69 | 98 | pc_aa_c40_2 | 0.38 | 0.54 | 0.25 |
| 33 | c18_2 | 0.61 | 1.55* | 0.98 | 99 | pc_aa_c40_6 | 0.97 | 0.68 | 0.72 |
| 34 | c2 | 0.55 | 0.68 | 0.29 | 100 | pc_ae_c36_0 | 1.18 | 0.88 | 1.43 |
| 35 | c3 | 1.03 | 1.03 | 0.29 | 101 | pc_ae_c40_6 | 0.78 | 0.77 | 0.73 |
| 36 | c3_1 | 0.31 | 1.00 | 0.66 | 102 | phenylalanine | 0.67 | 0.31 | 0.33 |
| 37 | c3oh | 0.50 | 1.36 | 0.97 | 103 | proline | 0.90 | 0.39 | 0.33 |
| 38 | c4 | 0.79 | 0.58 | 0.65 | 104 | propionic_acid | 0.95 | 1.61* | 2.17* |
| 39 | c4_1 | 0.91 | 1.61 | 0.82 | 105 | putrescine | 1.37 | 0.33 | 1.34* |
| 40 | c4oh | 0.53 | 0.55 | 0.37 | 106 | pyruvic_acid | 0.57 | 1.27 | 1.55 |
| 41 | c5 | 1.18 | 0.99 | 1.64* | 107 | sarcosine | 2.39* | 0.49 | 1.97* |
| 42 | c5_1 | 0.61 | 0.87 | 0.74 | 108 | serine | 0.80 | 1.04 | 1.15 |
| 43 | c5_1dc | 0.82 | 0.95 | 0.27 | 109 | serotonin | 0.58 | 0.97 | 0.87 |
| 44 | c5dc | 0.81 | 1.17 | 0.66 | 110 | sm_c16_0 | 0.63 | 0.94 | 0.95 |
| 45 | c5mdc | 1.23 | 1.21 | 0.28 | 111 | sm_c16_1 | 0.32 | 1.13 | 0.90 |
| 46 | c5oh | 1.06 | 0.80 | 1.01 | 112 | sm_c18_0 | 0.48 | 0.70 | 0.69 |
| 47 | c6 | 0.80 | 0.61 | 0.90 | 113 | sm_c18_1 | 0.51 | 0.87 | 0.72 |
| 48 | c6_1 | 0.77 | 1.09 | 0.70 | 114 | sm_c20_2 | 1.89 | 1.84 | 0.28 |
| 49 | c7dc | 0.62 | 1.28 | 1.11 | 115 | sm_oh__c14_1 | 0.86 | 0.92 | 0.79 |
| 50 | c8 | 0.63 | 0.64 | 0.74 | 116 | sm_oh__c16_1 | 0.63 | 0.97 | 0.46 |
| 51 | c9 | 1.87 | 1.85 | 0.58 | 117 | sm_oh__c22_1 | 1.44 | 0.70 | 1.20 |
| 52 | choline | 1.08 | 2.06 | 0.92 | 118 | sm_oh__c22_2 | 0.82 | 0.81 | 0.62 |
| 53 | citric_acid | 0.51 | 1.43 | 0.65 | 119 | sm_oh__c24_1 | 0.83 | 0.84 | 0.96 |
| 54 | citrulline | 0.23 | 0.65 | 0.60 | 120 | spermidine | 0.28 | 1.56 | 1.34 |
| 55 | creatine | 0.49 | 0.47 | 0.51 | 121 | spermine | 0.73 | 2.08* | 1.39 |
| 56 | creatinine | 0.59 | 1.12 | 0.94 | 122 | succinic_acid | 0.69 | 1.96* | 1.17 |
| 57 | fumaric_acid | 0.32 | 0.94 | 0.58 | 123 | taurine | 1.44 | 1.93* | 0.58 |
| 58 | glucose | 0.54 | 0.85 | 1.02 | 124 | threonine | 0.56 | 0.76 | 0.47 |
| 59 | glutamic_acid | 0.64 | 0.89 | 1.17 | 125 | total_dimethylarginine | 0.60 | 1.27 | 1.25 |
| 60 | glutamine | 0.58 | 0.73 | 0.95 | 126 | trans_hydroxyproline | 1.65 | 0.55 | 1.66 |
| 61 | glycine | 0.38 | 0.88 | 0.34 | 127 | trimethylamine_n_oxide | 0.61 | 0.66 | 0.87 |
| 62 | hippuric_acid | 0.66 | 1.68 | 1.19 | 128 | tryptophan | 0.59 | 0.86 | 0.60 |
| 63 | histidine | 1.59 | 0.39 | 1.09 | 129 | tyrosine | 0.46 | 0.64 | 0.25 |
| 64 | homocysteine | 0.37 | 0.47 | 0.16 | 130 | uric_acid | 0.99 | 1.09 | 0.37 |
| 65 | homovanillic_acid | 1.53 | 0.38 | 1.17 | 131 | valine | 1.11 | 0.76 | 1.35 |
| 66 | indole_acetic_acid | 0.76 | 1.03 | 1.41 |  |  |  |  |  |

**Table S3a.** Proportions of biomarkers were found by our selection method over 1,000 bootstrap samples. Partial least square, fold-change, adjusted p-value, and elastic-net penalized logistic regression models were employed for comparing two groups between bacterial pneumonia-induced ARDS patients admitted to ICU (PNA/A) and H1N1-induced ARDS patients admitted to ICU (H1N1/A), between SARS-CoV-2 induced ARDS patients admitted to ICU (C19/A) and H1N1/A and between C19/A and PNA/A.

|  | Name | PNA/A vs H1N1/A | C19/A vs H1N1/A | C19/A vs PNA/A |  | Name | PNA/A vs H1N1/A | C19/A vs H1N1/A | C19/A vs PNA/A |
| --- | --- | --- | --- | --- | --- | --- | --- | --- | --- |
| 1 | acetyl_ornithine | 0.15 | 0.00 | 0.04 | 67 | isobutyric_acid | 0.80 | 0.11 | 0.92 |
| 2 | alanine | 0.18 | 0.65 | 0.07 | 68 | isoleucine | 0.04 | 0.02 | 0.28 |
| 3 | alpha_aminoadipic_acid | 0.00 | 0.69 | 0.47 | 69 | kynurenine | 0.14 | 0.05 | 0.00 |
| 4 | alpha_ketoglutaric_acid | 0.17 | 0.60 | 0.99 | 70 | lactic_acid | 0.00 | 0.98 | 0.82 |
| 5 | arginine | 0.03 | 0.70 | 0.73 | 71 | leucine | 0.00 | 0.35 | 0.06 |
| 6 | asparagine | 0.63 | 0.00 | 0.20 | 72 | lysine | 0.38 | 0.00 | 0.22 |
| 7 | aspartic_acid | 0.14 | 0.03 | 0.09 | 73 | lysopc_a_c14_0 | 0.05 | 0.00 | 0.01 |
| 8 | asymmetric_dimethylarginine | 0.02 | 0.59 | 0.74 | 74 | lysopc_a_c16_0 | 0.77 | 0.34 | 0.99 |
| 9 | beta_hydroxybutyric_acid | 0.01 | 0.00 | 0.00 | 75 | lysopc_a_c16_1 | 0.25 | 0.00 | 0.16 |
| 0 | betaine | 0.32 | 0.00 | 0.20 | 76 | lysopc_a_c17_0 | 0.79 | 0.07 | 1.00 |
| 11 | butyric_acid | 0.66 | 0.13 | 0.57 | 77 | lysopc_a_c18_0 | 1.00 | 0.11 | 1.00 |
| 12 | c0 | 0.08 | 0.00 | 0.03 | 78 | lysopc_a_c18_1 | 0.99 | 0.03 | 1.00 |
| 13 | c10 | 0.03 | 0.14 | 0.01 | 79 | lysopc_a_c18_2 | 0.74 | 0.02 | 0.82 |
| 14 | c10_1 | 0.01 | 0.49 | 0.15 | 80 | lysopc_a_c20_3 | 0.53 | 0.00 | 0.22 |
| 15 | c10_2 | 0.48 | 0.22 | 0.00 | 81 | lysopc_a_c20_4 | 0.90 | 0.11 | 0.92 |
| 16 | c12 | 0.01 | 0.05 | 0.14 | 82 | lysopc_a_c24_0 | 0.46 | 0.05 | 0.03 |
| 17 | c12_1 | 0.00 | 0.01 | 0.01 | 83 | lysopc_a_c26_0 | 0.00 | 0.00 | 0.01 |
| 18 | c12dc | 0.79 | 0.03 | 0.73 | 84 | lysopc_a_c26_1 | 0.42 | 0.15 | 0.01 |
| 19 | c14 | 0.00 | 0.69 | 0.63 | 85 | lysopc_a_c28_0 | 0.00 | 0.50 | 0.30 |
| 20 | c14_1 | 0.61 | 0.23 | 0.01 | 86 | lysopc_a_c28_1 | 0.02 | 0.04 | 0.00 |
| 21 | c14_1oh | 0.13 | 0.00 | 0.34 | 87 | methionine | 0.16 | 0.00 | 0.06 |
| 22 | c14_2 | 0.03 | 0.17 | 0.01 | 88 | methionine_sulfoxide | 0.06 | 0.05 | 0.33 |
| 23 | c14_2oh | 0.80 | 0.35 | 0.30 | 89 | methylhistidine | 0.00 | 0.11 | 0.07 |
| 24 | c16 | 0.08 | 0.71 | 0.40 | 90 | methylmalonic_acid | 0.02 | 0.02 | 0.25 |
| 25 | c16_1 | 0.63 | 0.34 | 0.01 | 91 | ornithine | 0.49 | 0.13 | 0.75 |
| 26 | c16_1oh | 0.12 | 0.00 | 0.05 | 92 | pc_aa_c32_2 | 0.26 | 0.58 | 0.02 |
| 27 | c16_2 | 0.76 | 0.00 | 0.79 | 93 | pc_aa_c36_0 | 0.05 | 0.56 | 0.06 |
| 28 | c16_2oh | 0.03 | 0.01 | 0.10 | 94 | pc_aa_c36_6 | 0.08 | 0.25 | 0.01 |
| 29 | c16oh | 0.13 | 0.66 | 0.05 | 95 | pc_aa_c38_0 | 0.05 | 0.00 | 0.02 |
| 30 | c18 | 0.12 | 0.77 | 0.08 | 96 | pc_aa_c38_6 | 0.01 | 0.00 | 0.00 |
| 31 | c18_1 | 0.06 | 0.88 | 0.39 | 97 | pc_aa_c40_1 | 0.42 | 0.01 | 0.60 |
| 32 | c18_1oh | 0.03 | 0.01 | 0.11 | 98 | pc_aa_c40_2 | 0.00 | 0.01 | 0.00 |
| 33 | c18_2 | 0.07 | 0.98 | 0.46 | 99 | pc_aa_c40_6 | 0.17 | 0.00 | 0.06 |
| 34 | c2 | 0.00 | 0.00 | 0.00 | 100 | pc_ae_c36_0 | 0.42 | 0.05 | 0.72 |
| 35 | c3 | 0.27 | 0.25 | 0.00 | 101 | pc_ae_c40_6 | 0.03 | 0.00 | 0.09 |
| 36 | c3_1 | 0.00 | 0.29 | 0.18 | 102 | phenylalanine | 0.03 | 0.00 | 0.00 |
| 37 | c3oh | 0.00 | 0.64 | 0.45 | 103 | proline | 0.07 | 0.00 | 0.00 |
| 38 | c4 | 0.07 | 0.00 | 0.10 | 104 | propionic_acid | 0.50 | 0.96 | 1.00 |
| 39 | c4_1 | 0.15 | 0.65 | 0.24 | 105 | putrescine | 0.66 | 0.00 | 0.95 |
| 40 | c4oh | 0.00 | 0.00 | 0.00 | 106 | pyruvic_acid | 0.06 | 0.63 | 0.77 |
| 41 | c5 | 0.62 | 0.30 | 0.97 | 107 | sarcosine | 1.00 | 0.01 | 1.00 |
| 42 | c5_1 | 0.01 | 0.03 | 0.00 | 108 | serine | 0.11 | 0.16 | 0.51 |
| 43 | c5_1dc | 0.10 | 0.40 | 0.00 | 109 | serotonin | 0.00 | 0.59 | 0.43 |
| 44 | c5dc | 0.06 | 0.17 | 0.00 | 110 | sm_c16_0 | 0.02 | 0.05 | 0.42 |
| 45 | c5mdc | 0.50 | 0.40 | 0.00 | 111 | sm_c16_1 | 0.00 | 0.39 | 0.37 |
| 46 | c5oh | 0.24 | 0.03 | 0.29 | 112 | sm_c18_0 | 0.00 | 0.00 | 0.00 |
| 47 | c6 | 0.10 | 0.06 | 0.28 | 113 | sm_c18_1 | 0.00 | 0.10 | 0.01 |
| 48 | c6_1 | 0.04 | 0.51 | 0.06 | 114 | sm_c20_2 | 0.78 | 0.79 | 0.00 |
| 49 | c7dc | 0.01 | 0.74 | 0.64 | 115 | sm_oh__c14_1 | 0.07 | 0.02 | 0.19 |
| 50 | c8 | 0.01 | 0.00 | 0.06 | 116 | sm_oh__c16_1 | 0.01 | 0.03 | 0.00 |
| 51 | c9 | 0.80 | 0.80 | 0.01 | 117 | sm_oh__c22_1 | 0.42 | 0.00 | 0.54 |
| 52 | choline | 0.52 | 0.80 | 0.25 | 118 | sm_oh__c22_2 | 0.05 | 0.00 | 0.01 |
| 53 | citric_acid | 0.16 | 0.39 | 0.23 | 119 | sm_oh__c24_1 | 0.06 | 0.02 | 0.33 |
| 54 | citrulline | 0.00 | 0.04 | 0.04 | 120 | spermidine | 0.00 | 0.80 | 0.80 |
| 55 | creatine | 0.02 | 0.03 | 0.15 | 121 | spermine | 0.49 | 0.95 | 0.83 |
| 56 | creatinine | 0.00 | 0.16 | 0.16 | 122 | succinic_acid | 0.15 | 1.00 | 0.75 |
| 57 | fumaric_acid | 0.00 | 0.31 | 0.08 | 123 | taurine | 0.85 | 0.99 | 0.08 |
| 58 | glucose | 0.52 | 0.06 | 0.48 | 124 | threonine | 0.00 | 0.14 | 0.04 |
| 59 | glutamic_acid | 0.02 | 0.16 | 0.54 | 125 | total_dimethylarginine | 0.00 | 0.42 | 0.55 |
| 60 | glutamine | 0.00 | 0.09 | 0.41 | 126 | trans_hydroxyproline | 0.84 | 0.05 | 0.84 |
| 61 | glycine | 0.00 | 0.06 | 0.00 | 127 | trimethylamine_n_oxide | 0.06 | 0.03 | 0.17 |
| 62 | hippuric_acid | 0.08 | 0.90 | 0.64 | 128 | tryptophan | 0.02 | 0.06 | 0.00 |
| 63 | histidine | 0.70 | 0.00 | 0.54 | 129 | tyrosine | 0.00 | 0.05 | 0.01 |
| 64 | homocysteine | 0.00 | 0.00 | 0.00 | 130 | uric_acid | 0.23 | 0.32 | 0.00 |
| 65 | homovanillic_acid | 0.71 | 0.01 | 0.75 | 131 | valine | 0.26 | 0.07 | 0.60 |
| 66 | indole_acetic_acid | 0.08 | 0.34 | 0.69 |  |  |  |  |  |

**Table S4.** Variable importance on the projection (VIP) scores was calculated by PLS-DA separating SARS-CoV-2 induced ARDS patients admitted to ICU (C19/A) and SARS-CoV-2 infected patients admitted to a hospital but not sick enough to be admitted to ICU (C19/P). * is a biomarker found by our selection method over 1,000 bootstrap samples.

|  | Name | C19/A vs  C19/P |  | Name | C19/A vs  C19/P |
| --- | --- | --- | --- | --- | --- |
| 1 | acetyl_ornithine | 2.59* | 67 | isobutyric_acid | 2.16 |
| 2 | alanine | 0.72 | 68 | isoleucine | 1.71 |
| 3 | alpha_aminoadipic_acid | 1.67 | 69 | kynurenine | 0.37 |
| 4 | alpha_ketoglutaric_acid | 0.86 | 70 | lactic_acid | 0.94 |
| 5 | arginine | 1.25 | 71 | leucine | 1.73 |
| 6 | asparagine | 1.00 | 72 | lysine | 1.39 |
| 7 | aspartic_acid | 0.37 | 73 | lysopc_a_c14_0 | 0.66 |
| 8 | asymmetric_dimethylarginine | 0.41 | 74 | lysopc_a_c16_0 | 0.90 |
| 9 | beta_hydroxybutyric_acid | 0.45 | 75 | lysopc_a_c16_1 | 0.60 |
| 0 | betaine | 0.70 | 76 | lysopc_a_c17_0 | 1.00 |
| 11 | butyric_acid | 1.77 | 77 | lysopc_a_c18_0 | 0.99 |
| 12 | c0 | 1.70 | 78 | lysopc_a_c18_1 | 0.76 |
| 13 | c10 | 1.16 | 79 | lysopc_a_c18_2 | 0.59 |
| 14 | c10_1 | 0.97 | 80 | lysopc_a_c20_3 | 0.29 |
| 15 | c10_2 | 0.49 | 81 | lysopc_a_c20_4 | 0.86 |
| 16 | c12 | 0.46 | 82 | lysopc_a_c24_0 | 1.09 |
| 17 | c12_1 | 0.96 | 83 | lysopc_a_c26_0 | 0.44 |
| 18 | c12dc | 1.89 | 84 | lysopc_a_c26_1 | 0.97 |
| 19 | c14 | 0.53 | 85 | lysopc_a_c28_0 | 0.38 |
| 20 | c14_1 | 0.65 | 86 | lysopc_a_c28_1 | 0.49 |
| 21 | c14_1oh | 0.29 | 87 | methionine | 1.30 |
| 22 | c14_2 | 0.65 | 88 | methionine_sulfoxide | 0.79 |
| 23 | c14_2oh | 1.28 | 89 | methylhistidine | 0.75 |
| 24 | c16 | 0.84 | 90 | methylmalonic_acid | 0.42 |
| 25 | c16_1 | 1.06 | 91 | ornithine | 0.76 |
| 26 | c16_1oh | 0.62 | 92 | pc_aa_c32_2 | 0.77 |
| 27 | c16_2 | 0.58 | 93 | pc_aa_c36_0 | 0.66 |
| 28 | c16_2oh | 0.53 | 94 | pc_aa_c36_6 | 0.48 |
| 29 | c16oh | 1.08 | 95 | pc_aa_c38_0 | 0.56 |
| 30 | c18 | 0.52 | 96 | pc_aa_c38_6 | 0.80 |
| 31 | c18_1 | 1.08 | 97 | pc_aa_c40_1 | 1.09 |
| 32 | c18_1oh | 0.31 | 98 | pc_aa_c40_2 | 1.10 |
| 33 | c18_2 | 1.04 | 99 | pc_aa_c40_6 | 0.68 |
| 34 | c2 | 0.37 | 100 | pc_ae_c36_0 | 0.75 |
| 35 | c3 | 1.97 | 101 | pc_ae_c40_6 | 0.32 |
| 36 | c3_1 | 0.64 | 102 | phenylalanine | 0.92 |
| 37 | c3oh | 0.69 | 103 | proline | 0.60 |
| 38 | c4 | 0.85 | 104 | propionic_acid | 1.66 |
| 39 | c4_1 | 0.61 | 105 | putrescine | 0.51 |
| 40 | c4oh | 0.71 | 106 | pyruvic_acid | 0.94 |
| 41 | c5 | 1.75 | 107 | sarcosine | 0.53 |
| 42 | c5_1 | 1.39 | 108 | serine | 0.94 |
| 43 | c5_1dc | 0.44 | 109 | serotonin | 1.38 |
| 44 | c5dc | 1.58 | 110 | sm_c16_0 | 0.83 |
| 45 | c5mdc | 1.22 | 111 | sm_c16_1 | 0.98 |
| 46 | c5oh | 2.23 | 112 | sm_c18_0 | 0.44 |
| 47 | c6 | 0.95 | 113 | sm_c18_1 | 0.56 |
| 48 | c6_1 | 0.60 | 114 | sm_c20_2 | 0.95 |
| 49 | c7dc | 0.65 | 115 | sm_oh__c14_1 | 0.74 |
| 50 | c8 | 0.51 | 116 | sm_oh__c16_1 | 0.68 |
| 51 | c9 | 1.16 | 117 | sm_oh__c22_1 | 0.60 |
| 52 | choline | 1.00 | 118 | sm_oh__c22_2 | 0.75 |
| 53 | citric_acid | 0.68 | 119 | sm_oh__c24_1 | 0.92 |
| 54 | citrulline | 0.81 | 120 | spermidine | 1.21 |
| 55 | creatine | 0.69 | 121 | spermine | 1.22 |
| 56 | creatinine | 0.76 | 122 | succinic_acid | 0.86 |
| 57 | fumaric_acid | 0.89 | 123 | taurine | 0.91 |
| 58 | glucose | 1.58 | 124 | threonine | 0.91 |
| 59 | glutamic_acid | 0.61 | 125 | total_dimethylarginine | 0.74 |
| 60 | glutamine | 0.82 | 126 | trans_hydroxyproline | 0.88 |
| 61 | glycine | 0.79 | 127 | trimethylamine_n_oxide | 0.76 |
| 62 | hippuric_acid | 0.81 | 128 | tryptophan | 0.87 |
| 63 | histidine | 0.48 | 129 | tyrosine | 1.19 |
| 64 | homocysteine | 0.57 | 130 | uric_acid | 0.85 |
| 65 | homovanillic_acid | 0.31 | 131 | valine | 1.69 |
| 66 | indole_acetic_acid | 1.60 |  |  |  |

**Table S4a.** Proportions of biomarkers were found by our selection method over 1,000 bootstrap samples. Partial least square, fold-change, adjusted p-value, and elastic-net penalized logistic regression models were employed for comparing two groups between SARS-CoV-2 induced ARDS patients admitted to ICU (C19/A) and SARS-CoV-2 infected patients admitted to a hospital but not sick enough to be admitted to ICU (C19/P).

|  | Name | C19/A vs  C19/P |  | Name | C19/A vs  C19/P |
| --- | --- | --- | --- | --- | --- |
| 1 | acetyl_ornithine | 0.98 | 67 | isobutyric_acid | 0.65 |
| 2 | alanine | 0.01 | 68 | isoleucine | 0.61 |
| 3 | alpha_aminoadipic_acid | 0.44 | 69 | kynurenine | 0.01 |
| 4 | alpha_ketoglutaric_acid | 0.05 | 70 | lactic_acid | 0.06 |
| 5 | arginine | 0.18 | 71 | leucine | 0.47 |
| 6 | asparagine | 0.10 | 72 | lysine | 0.60 |
| 7 | aspartic_acid | 0.02 | 73 | lysopc_a_c14_0 | 0.03 |
| 8 | asymmetric_dimethylarginine | 0.02 | 74 | lysopc_a_c16_0 | 0.04 |
| 9 | beta_hydroxybutyric_acid | 0.02 | 75 | lysopc_a_c16_1 | 0.01 |
| 0 | betaine | 0.12 | 76 | lysopc_a_c17_0 | 0.09 |
| 11 | butyric_acid | 0.44 | 77 | lysopc_a_c18_0 | 0.08 |
| 12 | c0 | 0.59 | 78 | lysopc_a_c18_1 | 0.01 |
| 13 | c10 | 0.27 | 79 | lysopc_a_c18_2 | 0.01 |
| 14 | c10_1 | 0.15 | 80 | lysopc_a_c20_3 | 0.00 |
| 15 | c10_2 | 0.01 | 81 | lysopc_a_c20_4 | 0.01 |
| 16 | c12 | 0.04 | 82 | lysopc_a_c24_0 | 0.30 |
| 17 | c12_1 | 0.12 | 83 | lysopc_a_c26_0 | 0.01 |
| 18 | c12dc | 0.56 | 84 | lysopc_a_c26_1 | 0.16 |
| 19 | c14 | 0.03 | 85 | lysopc_a_c28_0 | 0.02 |
| 20 | c14_1 | 0.07 | 86 | lysopc_a_c28_1 | 0.01 |
| 21 | c14_1oh | 0.02 | 87 | methionine | 0.20 |
| 22 | c14_2 | 0.10 | 88 | methionine_sulfoxide | 0.05 |
| 23 | c14_2oh | 0.32 | 89 | methylhistidine | 0.06 |
| 24 | c16 | 0.04 | 90 | methylmalonic_acid | 0.00 |
| 25 | c16_1 | 0.22 | 91 | ornithine | 0.00 |
| 26 | c16_1oh | 0.02 | 92 | pc_aa_c32_2 | 0.03 |
| 27 | c16_2 | 0.07 | 93 | pc_aa_c36_0 | 0.02 |
| 28 | c16_2oh | 0.02 | 94 | pc_aa_c36_6 | 0.01 |
| 29 | c16oh | 0.27 | 95 | pc_aa_c38_0 | 0.02 |
| 30 | c18 | 0.00 | 96 | pc_aa_c38_6 | 0.12 |
| 31 | c18_1 | 0.19 | 97 | pc_aa_c40_1 | 0.22 |
| 32 | c18_1oh | 0.03 | 98 | pc_aa_c40_2 | 0.24 |
| 33 | c18_2 | 0.19 | 99 | pc_aa_c40_6 | 0.09 |
| 34 | c2 | 0.00 | 100 | pc_ae_c36_0 | 0.03 |
| 35 | c3 | 0.44 | 101 | pc_ae_c40_6 | 0.01 |
| 36 | c3_1 | 0.07 | 102 | phenylalanine | 0.24 |
| 37 | c3oh | 0.00 | 103 | proline | 0.03 |
| 38 | c4 | 0.07 | 104 | propionic_acid | 0.62 |
| 39 | c4_1 | 0.01 | 105 | putrescine | 0.02 |
| 40 | c4oh | 0.10 | 106 | pyruvic_acid | 0.11 |
| 41 | c5 | 0.79 | 107 | sarcosine | 0.04 |
| 42 | c5_1 | 0.32 | 108 | serine | 0.05 |
| 43 | c5_1dc | 0.01 | 109 | serotonin | 0.36 |
| 44 | c5dc | 0.24 | 110 | sm_c16_0 | 0.06 |
| 45 | c5mdc | 0.11 | 111 | sm_c16_1 | 0.06 |
| 46 | c5oh | 0.73 | 112 | sm_c18_0 | 0.01 |
| 47 | c6 | 0.21 | 113 | sm_c18_1 | 0.05 |
| 48 | c6_1 | 0.00 | 114 | sm_c20_2 | 0.06 |
| 49 | c7dc | 0.05 | 115 | sm_oh__c14_1 | 0.02 |
| 50 | c8 | 0.00 | 116 | sm_oh__c16_1 | 0.03 |
| 51 | c9 | 0.38 | 117 | sm_oh__c22_1 | 0.01 |
| 52 | choline | 0.19 | 118 | sm_oh__c22_2 | 0.04 |
| 53 | citric_acid | 0.17 | 119 | sm_oh__c24_1 | 0.08 |
| 54 | citrulline | 0.09 | 120 | spermidine | 0.14 |
| 55 | creatine | 0.10 | 121 | spermine | 0.19 |
| 56 | creatinine | 0.04 | 122 | succinic_acid | 0.05 |
| 57 | fumaric_acid | 0.05 | 123 | taurine | 0.06 |
| 58 | glucose | 0.29 | 124 | threonine | 0.03 |
| 59 | glutamic_acid | 0.03 | 125 | total_dimethylarginine | 0.07 |
| 60 | glutamine | 0.08 | 126 | trans_hydroxyproline | 0.15 |
| 61 | glycine | 0.06 | 127 | trimethylamine_n_oxide | 0.06 |
| 62 | hippuric_acid | 0.08 | 128 | tryptophan | 0.08 |
| 63 | histidine | 0.04 | 129 | tyrosine | 0.17 |
| 64 | homocysteine | 0.04 | 130 | uric_acid | 0.14 |
| 65 | homovanillic_acid | 0.01 | 131 | valine | 0.32 |
| 66 | indole_acetic_acid | 0.51 |  |  |  |

**Table S5.** Means and Standard Deviation of SARS-CoV-2 induced ARDS patients admitted to ICU (C19/A) and Validation group corresponding to C19/A (C19/AV).

|  | Group | C19/A | | C19/AV | |  | Group | C19/A | | C19/AV | |
| --- | --- | --- | --- | --- | --- | --- | --- | --- | --- | --- | --- |
|  | Name | Mean | SD | Mean | SD |  | Name | Mean | SD | Mean | SD |
| 1 | acetyl_ornithine | 0.448 | 0.415 | 0.447 | 0.275 | 67 | isobutyric_acid | 0.521 | 0.433 | 0.927 | 0.495 |
| 2 | alanine | 355.785 | 145.931 | 266.739 | 123.948 | 68 | isoleucine | 58.52 | 16.745 | 61.325 | 18.549 |
| 3 | alpha_aminoadipic_acid | 1.16 | 0.686 | 0.802 | 0.462 | 69 | kynurenine | 4.011 | 2.52 | 3.728 | 2.675 |
| 4 | alpha_ketoglutaric_acid | 29.744 | 16.715 | 12.913 | 6.956 | 70 | lactic_acid | 5006.046 | 2118.964 | 1453.627 | 561.587 |
| 5 | arginine | 24.516 | 9.987 | 43.741 | 13.257 | 71 | leucine | 143.485 | 50.377 | 146.163 | 49.321 |
| 6 | asparagine | 29.469 | 11.818 | 34.823 | 10.537 | 72 | lysine | 115.382 | 24.197 | 150.425 | 53.794 |
| 7 | aspartic_acid | 10.719 | 7.084 | 5.415 | 4.641 | 73 | lysopc_a_c14_0 | 1.156 | 0.48 | 0.901 | 0.232 |
| 8 | asymmetric_dimethylarginine | 0.331 | 0.069 | 0.403 | 0.062 | 74 | lysopc_a_c16_0 | 91.224 | 39.3 | 48.628 | 13.44 |
| 9 | beta_hydroxybutyric_acid | 416.358 | 435.044 | 519.309 | 510.563 | 75 | lysopc_a_c16_1 | 1.251 | 0.489 | 1.175 | 0.467 |
| 0 | betaine | 27.275 | 14.799 | 40.984 | 34.95 | 76 | lysopc_a_c17_0 | 1.122 | 0.489 | 0.565 | 0.138 |
| 11 | butyric_acid | 0.743 | 0.817 | 0.628 | 0.203 | 77 | lysopc_a_c18_0 | 22.536 | 11.46 | 9.552 | 3.023 |
| 12 | c0 | 43.75 | 16.7 | 55.828 | 29.194 | 78 | lysopc_a_c18_1 | 13.188 | 4.605 | 8.545 | 4.475 |
| 13 | c10 | 0.244 | 0.18 | 0.283 | 0.101 | 79 | lysopc_a_c18_2 | 10.159 | 4.991 | 10.528 | 8.716 |
| 14 | c10_1 | 0.186 | 0.101 | 0.236 | 0.086 | 80 | lysopc_a_c20_3 | 2.557 | 0.624 | 3.66 | 1.589 |
| 15 | c10_2 | 0.064 | 0.033 | 0.051 | 0.016 | 81 | lysopc_a_c20_4 | 6.197 | 3.853 | 4.311 | 3.303 |
| 16 | c12 | 0.107 | 0.075 | 0.085 | 0.021 | 82 | lysopc_a_c24_0 | 0.071 | 0.02 | 0.073 | 0.02 |
| 17 | c12_1 | 0.118 | 0.075 | 0.137 | 0.096 | 83 | lysopc_a_c26_0 | 0.113 | 0.029 | 0.142 | 0.03 |
| 18 | c12dc | 0.015 | 0.003 | 0.014 | 0.004 | 84 | lysopc_a_c26_1 | 0.066 | 0.019 | 0.066 | 0.021 |
| 19 | c14 | 0.062 | 0.027 | 0.065 | 0.03 | 85 | lysopc_a_c28_0 | 0.114 | 0.026 | 0.149 | 0.032 |
| 20 | c14_1 | 0.1 | 0.075 | 0.126 | 0.052 | 86 | lysopc_a_c28_1 | 0.109 | 0.022 | 0.116 | 0.027 |
| 21 | c14_1oh | 0.022 | 0.005 | 0.02 | 0.006 | 87 | methionine | 21.056 | 8.958 | 29.301 | 13.443 |
| 22 | c14_2 | 0.044 | 0.025 | 0.066 | 0.04 | 88 | methionine_sulfoxide | 0.724 | 0.429 | 0.796 | 0.263 |
| 23 | c14_2oh | 0.017 | 0.005 | 0.017 | 0.005 | 89 | methylhistidine | 12.48 | 19.544 | 13.474 | 26.913 |
| 24 | c16 | 0.166 | 0.054 | 0.119 | 0.029 | 90 | methylmalonic_acid | 0.154 | 0.091 | 0.174 | 0.171 |
| 25 | c16_1 | 0.06 | 0.02 | 0.07 | 0.025 | 91 | ornithine | 83.331 | 19.207 | 58.351 | 23.047 |
| 26 | c16_1oh | 0.017 | 0.004 | 0.018 | 0.008 | 92 | pc_aa_c32_2 | 1.342 | 0.341 | 1.432 | 0.372 |
| 27 | c16_2 | 0.019 | 0.005 | 0.015 | 0.006 | 93 | pc_aa_c36_0 | 3.824 | 1.142 | 5.531 | 1.825 |
| 28 | c16_2oh | 0.011 | 0.003 | 0.014 | 0.006 | 94 | pc_aa_c36_6 | 0.335 | 0.212 | 0.345 | 0.092 |
| 29 | c16oh | 0.016 | 0.004 | 0.015 | 0.005 | 95 | pc_aa_c38_0 | 1.543 | 0.425 | 2.229 | 0.786 |
| 30 | c18 | 0.057 | 0.02 | 0.064 | 0.028 | 96 | pc_aa_c38_6 | 42.612 | 18.355 | 68.544 | 27.81 |
| 31 | c18_1 | 0.254 | 0.109 | 0.138 | 0.061 | 97 | pc_aa_c40_1 | 0.15 | 0.059 | 0.226 | 0.066 |
| 32 | c18_1oh | 0.013 | 0.003 | 0.02 | 0.009 | 98 | pc_aa_c40_2 | 0.224 | 0.105 | 0.236 | 0.077 |
| 33 | c18_2 | 0.091 | 0.035 | 0.066 | 0.035 | 99 | pc_aa_c40_6 | 11.839 | 4.257 | 21.522 | 9.529 |
| 34 | c2 | 12.437 | 5.275 | 12.201 | 6.441 | 100 | pc_ae_c36_0 | 1.186 | 0.345 | 1.674 | 0.383 |
| 35 | c3 | 0.418 | 0.242 | 0.463 | 0.17 | 101 | pc_ae_c40_6 | 1.938 | 0.657 | 2.749 | 0.781 |
| 36 | c3_1 | 0.027 | 0.009 | 0.042 | 0.021 | 102 | phenylalanine | 95.329 | 31.036 | 89.915 | 30.793 |
| 37 | c3oh | 0.024 | 0.005 | 0.032 | 0.013 | 103 | proline | 135.647 | 40.5 | 121.842 | 40.001 |
| 38 | c4 | 0.363 | 0.281 | 0.361 | 0.322 | 104 | propionic_acid | 0.743 | 0.434 | 2.19 | 1.571 |
| 39 | c4_1 | 0.023 | 0.009 | 0.034 | 0.012 | 105 | putrescine | 0.114 | 0.026 | 0.14 | 0.056 |
| 40 | c4oh | 0.116 | 0.065 | 0.136 | 0.128 | 106 | pyruvic_acid | 127.377 | 66.426 | 64.663 | 20.105 |
| 41 | c5 | 0.275 | 0.18 | 0.128 | 0.086 | 107 | sarcosine | 5.313 | 3.262 | 4.929 | 2.28 |
| 42 | c5_1 | 0.035 | 0.027 | 0.029 | 0.012 | 108 | serine | 80.823 | 24.956 | 72.478 | 19.883 |
| 43 | c5_1dc | 0.018 | 0.005 | 0.025 | 0.014 | 109 | serotonin | 0.089 | 0.087 | 0.049 | 0.039 |
| 44 | c5dc | 0.036 | 0.018 | 0.038 | 0.016 | 110 | sm_c16_0 | 93.215 | 19.537 | 122.75 | 28.972 |
| 45 | c5mdc | 0.037 | 0.023 | 0.045 | 0.024 | 111 | sm_c16_1 | 12.069 | 3.104 | 14.848 | 3.705 |
| 46 | c5oh | 0.042 | 0.027 | 0.027 | 0.014 | 112 | sm_c18_0 | 22.61 | 6.758 | 27.863 | 11.853 |
| 47 | c6 | 0.099 | 0.06 | 0.145 | 0.134 | 113 | sm_c18_1 | 8.773 | 2.513 | 10.524 | 4.378 |
| 48 | c6_1 | 0.026 | 0.009 | 0.037 | 0.021 | 114 | sm_c20_2 | 0.244 | 0.073 | 0.251 | 0.076 |
| 49 | c7dc | 0.039 | 0.023 | 0.071 | 0.033 | 115 | sm_oh__c14_1 | 4.086 | 1.059 | 5.174 | 1.373 |
| 50 | c8 | 0.14 | 0.106 | 0.199 | 0.105 | 116 | sm_oh__c16_1 | 2.776 | 0.721 | 3.008 | 0.917 |
| 51 | c9 | 0.051 | 0.024 | 0.083 | 0.068 | 117 | sm_oh__c22_1 | 9.091 | 2.343 | 7.84 | 2.146 |
| 52 | choline | 17.864 | 10.578 | 15.129 | 7.9 | 118 | sm_oh__c22_2 | 6.977 | 1.616 | 6.962 | 2.189 |
| 53 | citric_acid | 1745.427 | 4687.043 | 58.988 | 32.549 | 119 | sm_oh__c24_1 | 1.624 | 0.447 | 1.449 | 0.38 |
| 54 | citrulline | 14.714 | 6.577 | 16.523 | 8.605 | 120 | spermidine | 0.338 | 0.549 | 0.047 | 0.027 |
| 55 | creatine | 33.886 | 25.381 | 24.397 | 20.29 | 121 | spermine | 0.815 | 1.082 | 0.084 | 0.044 |
| 56 | creatinine | 121.187 | 173.789 | 139.571 | 220.224 | 122 | succinic_acid | 10.614 | 7.922 | 3.527 | 1.225 |
| 57 | fumaric_acid | 3.584 | 3.55 | 1.79 | 0.834 | 123 | taurine | 88.993 | 55.931 | 31.423 | 12.86 |
| 58 | glucose | 5682.503 | 5867.689 | 10316.22 | 4905.934 | 124 | threonine | 97.261 | 29.281 | 95.938 | 44.405 |
| 59 | glutamic_acid | 123.409 | 60.168 | 84.861 | 35.393 | 125 | total_dimethylarginine | 1.718 | 1.413 | 1.748 | 0.823 |
| 60 | glutamine | 329.522 | 125.922 | 434.272 | 133.465 | 126 | trans_hydroxyproline | 4.404 | 3.061 | 4.756 | 2.525 |
| 61 | glycine | 175.844 | 94.564 | 147.932 | 38.92 | 127 | trimethylamine_n_oxide | 4.366 | 9.973 | 3.446 | 10.367 |
| 62 | hippuric_acid | 4.6 | 9.818 | 18.269 | 26.659 | 128 | tryptophan | 27.201 | 8.639 | 23.832 | 9 |
| 63 | histidine | 58.596 | 10.344 | 58.252 | 10.262 | 129 | tyrosine | 71.208 | 17.394 | 68.824 | 21.389 |
| 64 | homocysteine | 7.773 | 1.852 | 8.838 | 3.8 | 130 | uric_acid | 248.537 | 123.593 | 181.739 | 75.141 |
| 65 | homovanillic_acid | 0.072 | 0.043 | 0.069 | 0.028 | 131 | valine | 248.806 | 67.233 | 218.378 | 50.497 |
| 66 | indole_acetic_acid | 0.645 | 0.996 | 0.842 | 0.765 |  |  |  |  |  |  |

**Figure S1.** A score plot is generated using PLS-DA in a pairwise manner between four study groups: non-ARDS ventilated control patients admitted to ICU (CTL), H1N1-induced ARDS patients admitted to ICU (H1N1/A), bacterial pneumonia-induced ARDS patients admitted to ICU (PNA/A), COVID-19-induced ARDS patients admitted to ICU (C19/A), and COVID-19 infected patients admitted to a hospital but not sick enough to be admitted to ICU (C19/P). Note that the second group is set as a reference for comparing two groups. Models are run with 25 patients per group.


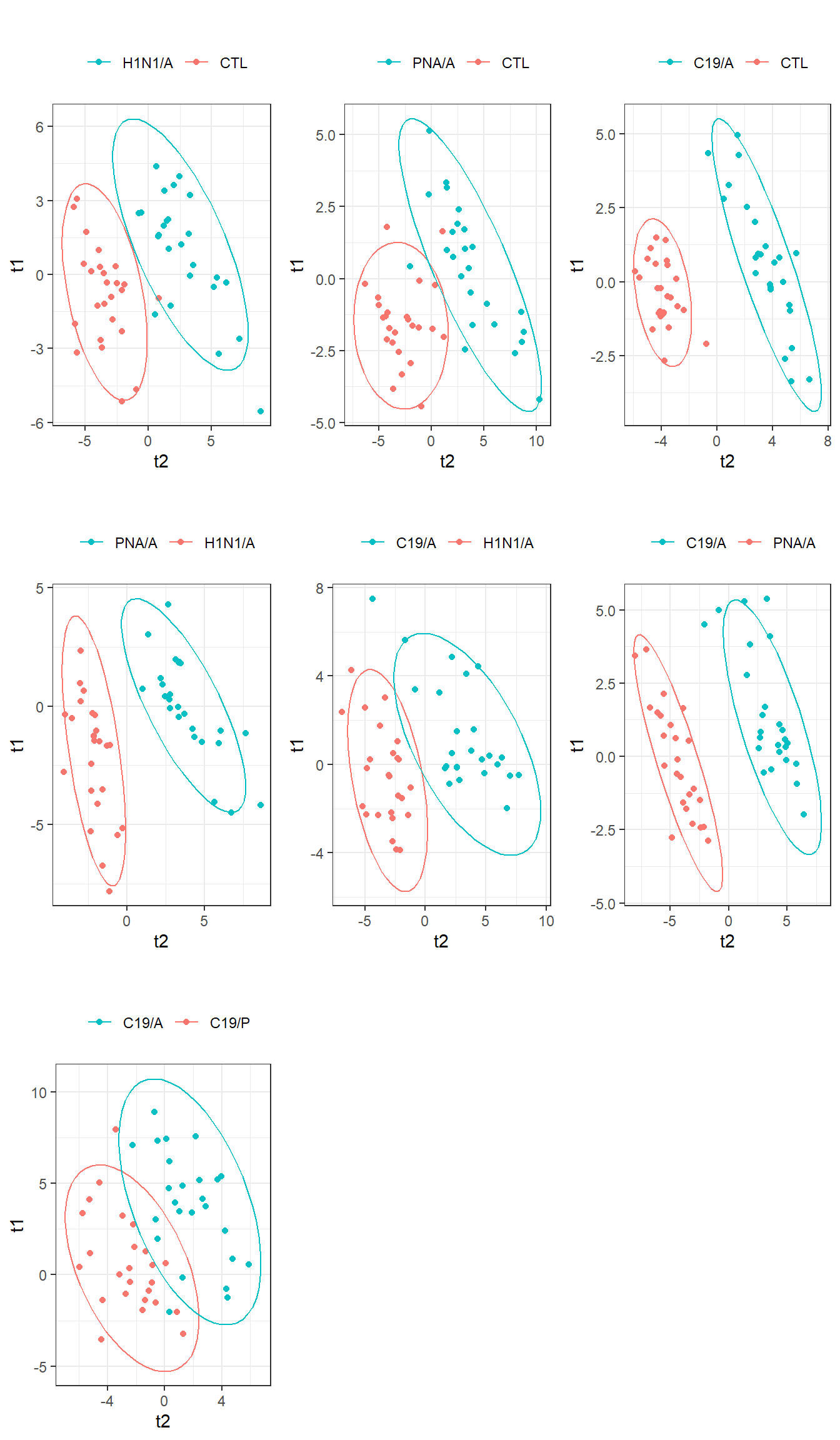


**Table S6.** The significant metabolites between the pairwise comparison of two cohorts using the non-parametric Wilcoxon method. The red box shows the increase of metabolites in the first cohort compared to the second. The blue box shows the decrease of metabolites in the first cohort compared to the second. P value <0.05 was considered significant.

|  | **C19/A vs. H1N1/A** | **C19/A vs. PNA/A** | **C19/A vs. CTR** | **C19/P vs. H1N1/A** | **C19/P vs. PNA/A** | **C19/P vs. CTR** | **H1N1/A vs. PNA/A** | **H1N1/A vs. CTR** | **PNA/A vs. CTR** |
| --- | --- | --- | --- | --- | --- | --- | --- | --- | --- |
| Acetyl-ornithine | ns | ns | ns | <.0001 | 0.0074 | 0.0074 | ns | ns | ns |
| Dimethylarginine | ns | 0.0002 | <.0001 | 0.0379 | <.0001 | <.0001 | ns | ns | ns |
| Alanine | <.0001 | 0.0179 | ns | <.0001 | 0.022 | ns | ns | <.0001 | 0.0313 |
| alpha-Aminoadipic acid | 0.0012 | 0.0012 | 0.0006 | ns | ns | ns | ns | ns | ns |
| alpha-Ketoglutaric acid | 0.0012 | <.0001 | <.0001 | 0.0017 | <.0001 | <.0001 | ns | ns | 0.0298 |
| Arginine | 0.0018 | <.0001 | <.0001 | 0.0001 | <.0001 | <.0001 | ns | <.0001 | 0.0123 |
| Asparagine | ns | ns | ns | ns | 0.003 | ns | 0.0028 | ns | 0.0232 |
| Aspartic acid | ns | ns | ns | ns | ns | 0.011 | 0.0189 | ns | ns |
| Betaine | ns | ns | ns | ns | ns | ns | ns | ns | ns |
| Butyric acid | ns | 0.0052 | ns | 0.0074 | ns | ns | 0.0002 | 0.0123 | 0.0123 |
| C0 | ns | ns | ns | 0.0328 | 0.0002 | 0.0025 | ns | ns | ns |
| C10:1 | 0.0436 | ns | 0.0179 | 0.0313 | ns | 0.0199 | ns | ns | ns |
| C10:2 | ns | ns | ns |  | ns |  | 0.0436 | ns | ns |
| C12DC | ns | 0.0019 | ns | 0.0043 | ns | 0.017 | 0.0009 | ns | 0.003 |
| C14 | 0.0022 | 0.0006 | ns | 0.0161 | 0.003 | ns | ns | 0.0117 | 0.001 |
| C14:1 | 0.0209 | ns | 0.0006 | 0.0036 | ns | ns | 0.0137 | <.0001 | 0.0016 |
| C14:2 | 0.0104 | ns | ns | 0.0062 | ns | ns | ns | 0.0001 | 0.0244 |
| C14:2OH | ns | 0.0436 | ns | 0.0008 | ns | 0.0161 | 0.0007 | ns | 0.0232 |
| C16 | <.0001 | 0.0083 | 0.0079 | <.0001 | 0.0209 | 0.0244 | ns | 0.0074 | ns |
| C16:1 | 0.0137 | ns | ns | 0.0017 | ns | ns | 0.0145 | 0.0117 | ns |
| C16:2 | ns | <.0001 | 0.022 | ns | 0.0036 | ns | 0.0093 | ns | ns |
| C16OH | 0.0093 | ns | ns | <.0001 | 0.0016 | 0.0004 | ns | ns | ns |
| C16:1OH | ns | ns | ns | ns | ns | ns | ns | ns | 0.0328 |
| C18 | <.0001 | ns | 0.0244 | <.0001 | ns | ns | 0.022 | 0.0145 | ns |
| C18:1 | <.0001 | 0.0041 | 0.0002 | <.0001 | <.0001 | <.0001 | ns | ns | ns |
| C18:2 | <.0001 | 0.002 | 0.0012 | <.0001 | 0.0003 | 0.0001 | 0.017 | 0.0244 | ns |
| C3 | 0.0379 | ns | 0.0046 | ns | 0.0013 | ns | 0.0145 | ns | 0.0003 |
| C3:1 | ns | ns | ns | 0.0015 | 0.001 | 0.0416 | ns | ns | ns |
| C3OH | 0.0397 | 0.0478 | 0.0036 | 0.0018 | 0.003 | 0.0004 | ns | ns | ns |
| C4 | ns | ns | 0.0079 | ns | 0.0016 | ns | 0.0397 | ns | <.0001 |
| C4:1 | 0.011 | ns | ns | 0.0013 | 0.0079 | ns | ns | ns | ns |
| C4OH | ns | ns | ns | 0.0478 | ns | ns | ns | ns | ns |
| C5 | 0.0436 | <.0001 | <.0001 | ns | ns | 0.003 | 0.0059 | 0.0001 | ns |
| C5:1 | ns | ns | ns | 0.0043 | ns | ns | ns | 0.013 | ns |
| C5:1DC | ns | ns | ns | 0.0137 | ns | 0.0052 | ns | ns | ns |
| C5DC | ns | ns | ns | 0.0117 | 0.013 | 0.0036 | ns | ns | ns |
| C5MDC | ns | ns | ns | ns | ns | ns | 0.0361 | ns | 0.0313 |
| C5OH | ns | ns | ns | 0.0004 | 0.0298 | 0.0006 | ns | ns | ns |
| C6 | ns | ns | ns | ns | 0.013 | 0.0004 | ns | ns | ns |
| C6:1 | 0.0328 | ns | ns | 0.0009 | 0.0043 | 0.0088 | ns | ns | ns |
| C7DC | 0.0137 | 0.0004 | ns | 0.0298 | 0.0004 | 0.0298 | ns | ns | 0.0298 |
| C8 |  |  | ns | ns | 0.0062 | ns | ns | ns | 0.001 |
| C9 | <.0001 | ns | ns | 0.0001 | ns | 0.013 | 0.0002 | <.0001 | ns |
| Choline | 0.0001 | 0.0457 | 0.0199 | 0.0117 | ns | ns | 0.0153 | 0.0478 | ns |
| Citric acid | ns | ns | 0.0043 | 0.0036 | ns | 0.0041 | 0.0344 | <.0001 | 0.0012 |
| Citrulline | ns | ns | 0.0002 | ns | ns | 0.0032 | ns | 0.0145 | 0.007 |
| Creatine | ns | ns | ns | ns | 0.0298 | ns | ns | ns | ns |
| Creatinine | ns | 0.0099 | ns | ns | ns | ns | ns | ns | ns |
| Fumaric acid | 0.0036 | 0.0436 | 0.002 | 0.0019 | 0.022 | 0.0004 | ns | ns | ns |
| Glucose |  | 0.0036 | 0.002 | <.0001 | <.0001 | ns | 0.001 | 0.0003 | ns |
| Glutamic acid | 0.0153 | 0.0008 | ns | ns | 0.0019 |  | ns | ns | 0.0043 |
| Glutamine | ns |  | 0.0006 | 0.0436 | 0.0153 | <.0001 | ns | 0.0088 | 0.0209 |
| Glycine | ns | ns | 0.003 | ns | ns | 0.0049 | ns | 0.0009 | 0.0179 |
| Hippuric acid | 0.0011 | 0.0016 |  | 0.0004 | 0.0023 | ns | ns | 0.0003 | 0.0179 |
| Histidine | ns | 0.0083 | 0.0043 | ns | 0.0002 | 0.0001 | 0.0015 | 0.0006 |  |
| Homocysteine | ns | ns | 0.0478 | ns | ns | 0.0123 | ns | ns | 0.0298 |
| Homovanillic acid | ns | 0.0008 | 0.0002 | ns | <.0001 | 0.0361 | 0.0041 | ns | <.0001 |
| Indole acetic acid | 0.0179 | 0.0001 |  | ns | 0.0179 | 0.0313 | ns | ns | ns |
| Isobutyric acid | ns | <.0001 | <.0001 | ns | 0.0032 | ns | <.0001 | 0.0001 | ns |
| Isoleucine | ns | 0.0244 | 0.027 | ns | ns | ns | ns | ns | ns |
| Kynurenine | ns | ns | <.0001 | ns | ns | 0.0001 | ns | <.0001 | 0.0005 |
| Lactic acid | <.0001 | <.0001 | <.0001 | <.0001 | <.0001 | <.0001 | ns | ns | ns |
| Leucine | 0.0099 | ns | 0.017 | ns | ns | ns | ns | ns | ns |
| Lysine | ns | ns | 0.0001 | 0.0189 | <.0001 | <.0001 | 0.0232 | <.0001 | ns |
| LysoPC a C14:0 | ns | ns | ns | ns | ns | 0.0436 | ns | 0.0153 | ns |
| LysoPC a C16:0 | 0.0006 | <.0001 | 0.0004 | 0.0046 | <.0001 | 0.0018 | <.0001 | ns | <.0001 |
| LysoPC a C16:1 |  | 0.011 | 0.0032 | ns | ns | 0.0014 | 0.0313 | 0.0038 | <.0001 |
| LysoPC a C17:0 | 0.022 | <.0001 | ns | 0.0028 | <.0001 | 0.0313 | <.0001 | ns | <.0001 |
| LysoPC a C18:0 | 0.0104 | <.0001 | 0.0022 | 0.0017 | <.0001 | 0.0002 | <.0001 | ns | <.0001 |
| LysoPC a C18:1 | ns | <.0001 | ns | ns | <.0001 | ns | <.0001 | ns | <.0001 |
| LysoPC a C18:2 | ns | <.0001 | ns | ns | <.0001 | ns | 0.0005 | 0.0397 | <.0001 |
| LysoPC a C20:3 | ns | ns | 0.0328 | ns | 0.027 | 0.0344 | 0.0093 | 0.013 | ns |
| LysoPC a C20:4 | 0.0361 | <.0001 | ns | ns | <.0001 | ns | 0.0002 | ns | <.0001 |
| LysoPC a C24:0 | ns | ns | 0.0004 | ns | 0.0257 | 0.0104 | 0.007 | 0.0036 | <.0001 |
| LysoPC a C26:0 | ns | ns | <.0001 | ns | ns | <.0001 |  | <.0001 | 0.0006 |
| LysoPC a C26:1 | ns | ns | 0.0062 | ns | ns | ns | 0.017 | ns | 0.0002 |
| LysoPC a C28:0 | 0.0244 | 0.0361 | <.0001 | 0.0328 | 0.0232 | <.0001 | ns | 0.0117 | 0.0052 |
| LysoPC a C28:1 | ns | ns | 0.0001 | ns | ns | 0.0002 | ns | 0.0028 | 0.0001 |
| Methionine | ns | ns | ns | ns | 0.0012 | ns | ns | ns | 0.0457 |
| Methionine-sulfoxide | ns | 0.0244 | ns | 0.0153 | 0.0011 | ns | ns | 0.0079 | 0.0002 |
| Methylhistidine | ns | ns | 0.0344 | ns | 0.0093 | ns | ns | ns | ns |
| Methylmalonic acid | ns | 0.0328 | 0.0457 | ns | ns | ns | ns | 0.0179 | 0.0007 |
| Ornithine | 0.0361 | <.0001 | <.0001 | ns | <.0001 | <.0001 | 0.0145 | 0.0018 | ns |
| PC aa C32:2 | 0.0153 | ns | ns | 0.0008 | ns | ns | ns | ns | ns |
| PC aa C36:0 | 0.0099 | ns | 0.0004 | 0.0014 | 0.0328 | 0.0003 | ns | ns | 0.0416 |
| PC aa C36:6 | 0.0079 | ns | 0.0001 | 0.011 | ns | <.0001 | 0.0478 | ns | 0.0015 |
| PC aa C38:0 | ns | ns | 0.007 | ns | ns | 0.007 | ns | 0.0034 | ns |
| PC aa C38:6 | ns | ns | 0.0036 | ns | 0.027 | <.0001 | ns | <.0001 | 0.0055 |
| PC aa C40:1 | ns | 0.0018 | 0.0028 | ns | 0.0153 | ns | 0.0041 | 0.013 | ns |
| PC aa C40:6 | ns | ns | 0.0006 | ns | 0.0059 | <.0001 |  | <.0001 | 0.0379 |
| PC ae C36:0 | ns | <.0001 | ns | 0.0361 | <.0001 | 0.027 | 0.0123 | ns | 0.0049 |
| PC ae C40:6 | ns | ns | <.0001 | ns | ns | <.0001 | ns | <.0001 | 0.0017 |
| Phenylalanine | ns | ns | <.0001 | ns | 0.0052 | ns | ns | 0.0055 | <.0001 |
| p-Hydroxyhippuric acid | ns | 0.0002 | 0.0478 | ns | <.0001 | 0.022 | <.0001 | 0.0016 | ns |
| Propionic acid | <.0001 | <.0001 | <.0001 | ns | <.0001 | <.0001 | 0.0002 | 0.0001 | ns |
| Putrescine | ns | <.0001 | 0.0038 | ns | <.0001 | 0.001 | 0.0009 | 0.0244 | ns |
| Pyruvic acid | 0.0003 | <.0001 | <.0001 | 0.0007 | <.0001 | 0.0002 | ns | <.0001 | ns |
| Sarcosine | ns | <.0001 | <.0001 | ns | <.0001 | <.0001 | <.0001 | ns | ns |
| Serotonin | ns | ns | ns | ns | ns | 0.0016 | ns | <.0001 | 0.0009 |
| Serine | 0.0283 | 0.0052 | ns | ns | 0.0232 | ns | ns | ns | 0.001 |
| SM C16:0 | ns | 0.0328 | ns | ns | ns | ns | ns | ns | 0.0283 |
| SM C16:1 | 0.0397 | 0.0361 | ns | 0.013 | 0.0049 | ns | ns | ns | ns |
| SM C18:1 | ns | ns | ns | 0.0436 | ns | ns | ns | ns | ns |
| SM C20:2 | <.0001 | ns | <.0001 | <.0001 | ns | 0.0003 | <.0001 | ns | 0.0008 |
| SM(OH) C14:1 | ns | ns | ns | ns | 0.0344 | ns | ns | ns | ns |
| SM(OH) C22:1 | ns | 0.0014 | ns | ns | 0.0006 | ns | 0.0066 | ns | 0.0043 |
| SM(OH) C22:2 | ns | ns | ns | ns | ns | ns | ns | ns | 0.0074 |
| SM(OH) C24:1 | 0.0379 | 0.0046 | ns | 0.0379 | 0.0022 | ns | ns | ns | ns |
| Spermidine | 0.0003 | 0.0001 | ns | <.0001 | <.0001 | 0.0004 | ns | ns | 0.0004 |
| Spermine | <.0001 | 0.0001 | 0.0002 | <.0001 | <.0001 | <.0001 | 0.001 | ns | ns |
| Succinic acid | <.0001 | 0.0001 | <.0001 | <.0001 | <.0001 | <.0001 | 0.0117 | ns | 0.0361 |
| Taurine | <.0001 | ns | 0.0099 | ns | 0.0079 | 0.001 | 0.0003 | <.0001 | ns |
| Dimethylarginine | 0.017 | 0.0015 | 0.0209 | 0.0436 | 0.0038 | ns | ns | 0.0003 | ns |
| Threonine | 0.0379 | ns | ns | ns | ns | 0.022 | ns | 0.011 | 0.0007 |
| trans-Hydroxyproline | ns | 0.0003 | 0.0017 | ns | 0.0006 | 0.0052 | 0.0011 | 0.0007 | 0.0478 |
| Tryptophan | ns | ns | 0.0244 | ns | ns | ns | ns | ns | ns |
| Valine | ns | 0.0017 | ns | ns | ns | ns | ns | ns | ns |
